# Platy-1 SINEs from Thirteen Diverse Genomes Reveal Callithrichidae Unique Amplification, Recent *Alouatta* Mobilization and Insights into Platyrrhine Phylogenetics

**DOI:** 10.3390/genes17010100

**Published:** 2026-01-19

**Authors:** Jessica M. Storer, Jerilyn A. Walker, Sarah O. Massey, Thomas O. Beckstrom, Mark A. Batzer

**Affiliations:** 1 Department of Biological Sciences, Louisiana State University, 202 Life Sciences Building, Baton Rouge, LA 70803, USA; jessica.storer@uconn.edu (J.M.S.); jawalker@lsu.edu (J.A.W.); smass2@lsuhsc.edu (S.O.M.); tbecks@uw.edu (T.O.B.); 2 Institute for Systems Biology, Seattle, WA 98109, USA; 3 Institute for Systems Genomics, University of Connecticut, Storrs, CT 06269, USA; 4 Department of Oral and Maxillofacial Surgery, University of Washington, 1959 NE Pacific Street, Health Sciences Building B-241, Seattle, WA 98195, USA

**Keywords:** platyrrhine, Callithrichidae, tamarin, Platy-1, *Saguinus*, MEI, SINE, phylogeny

## Abstract

Background/Objectives: In 2023, we reported that the tamarins (genus *Saguinus*) *Saguinus imperator* and *Saguinus midas* have had an extensive independent expansion of Platy-1 SINEs compared to previously characterized platyrrhine genomes among traditional cebids. This study investigates the amplification dynamics of Platy-1 insertions across thirteen diverse genomes representing each Platyrrhini family, including two from Pitheciidae and three from Atelidae. Methods: By comparing the distribution of Platy-1 subfamily content, total interspersed repeat content and the proximity of Platy-1 insertions to, or within, other repeats across evolutionary taxa, this study begins to identify genomic landscape features that are unique to family Callithrichidae that correlate with LINE (L1). Results: Platy-1 radiation in non-callithrichid taxa derives primarily from older subfamilies 1-4, 1-4a (as reported here for genus *Alouatta*) and 1-5, whereas callithrichids proliferate higher numbers of Platy-1 copies via independent bursts from much younger sources. Linage-specific Platy-1 activity was notable in two of the new genomes studied, Bolivian titi and mantled howler monkey, both with a relatively low copy number. Variable presence/absence patterns across evolutionary taxa support the traditional platyrrhine branching order Pitheciidae–Atelidae–Cebidae. Only one Platy-1-4a insertion polymorphism placed Aotidae between Atelidae and Cebidae, as opposed to between Cebidae and Callithrichidae. Conclusions: This study shows that callithrichids, and *Saguinus* tamarins in particular, are unique among platyrrhines with regard to their extensive rate of Platy-1 mobilization, a dynamic that appears to be correlated with LINE (L1) genomic content. *Alouatta* has two young lineage-specific Platy-1 subfamilies. With strong evidence of incomplete lineage sorting (ILS) and rapid radiation, the accurate placement of *Aotus* remains elusive.

## 1. Introduction

Platy-1 retroposons are ~100 base pair (bp) short interspersed elements (SINEs) that are unique to platyrrhine primates [1,2,3]. They mobilize via a non-autonomous “copy and paste” mechanism through an RNA intermediate, similarly to primate-specific *Alu* SINEs, utilizing the autonomous LINE (L1) enzymatic machinery. This mobilization process is termed “target-primed reverse transcription” (TPRT) [4,5]. Recently, we characterized these Platy-1 mobile element insertions (MEIs) in two tamarin species (genus *Saguinus*), *S. imperator* and *S. midas*, illustrating that each lineage has extensive independent expansion of Platy-1 elements from six new lineage-specific subfamilies, dominated primarily by the youngest subfamily, Platy-1-8c_*Saguinus* [3]. With over 11,000 full-length Platy-1 elements in each of these two *Saguinus* genomes, their copy numbers far exceeded the roughly 2200 initially reported in the common marmoset genome (*Callithrix jacchus*) [1], another member of the Callithrichidae family of platyrrhine primates. These platyrrhine-specific SINEs have now been characterized in six species, including two members of the Cebidae family—the squirrel monkey (*Saimiri boliviensis*; saiBol1) and capuchin monkey (*Cebus imitator*; Cebus_imitator-1.0)—along with the owl monkey (*Aotus nancymaae*; Anan_1.0) from family Aotidae [2]. Thus far, the bulk of Platy-1 mobilization activity has been observed among Callithrichidae species (marmoset and tamarins), with only limited lineage-specific amplification evident in *Aotus*, while being virtually quiescent in the *Saimiri* and *Cebus* lineages [2].

The purpose of this study was to expand the investigation of Platy-1 MEIs to include representatives of all platyrrhine families, including Pitheciidae and Atelidae, to identify differences across taxa and determine if other lineages mirror the proliferation observed in tamarins. Thus far, no species other than traditional cebids have been analyzed for Platy-1 content. Whole genome sequences (WGSs) for a total of thirteen species, including *S. imperator*, *S. midas* and *C. jacchus* from [3] were analyzed in this study. Two additional assemblies representing the Cebidae family (*Cebus albifrons*, white-fronted capuchin and *Sapajus apella*, tufted capuchin) are included, as well as two from family Pitheciidae (*Pithecia pithecia*, white-faced saki and *Plecturocebus donacophilus*, Bolivian titi) and three from family Atelidae (*Alouatta palliata*, mantled howler monkey, *Ateles geoffroyi*, black-handed spider monkey and *Ateles hybridus*, brown spider monkey). Each was compared to previously characterized species representing Cebidae, Aotidae and Callithrichidae, while implementing more recent genome assemblies. The data reported in this study suggest that the amplification dynamics of TPRT-integrated Platy-1 retroposons in Callithrichidae are unique thus far among platyrrhine primates and appear to be correlated to the number of long interspersed elements (LINE) L1. Further, the amplification burst observed in *Saguinus* tamarins [3] represents an unprecedented SINE expansion among platyrrhine primates. Future investigations should focus on potential catalysts and the evolutionary impact of Platy-1 and L1 mobilization on the genomic structural variation in tamarins.

## 2. Materials and Methods

### 2.1. Full Length Platy-1 Elements

Whole genome sequence (WGS) assemblies for thirteen species representing all platyrrhine families were downloaded from the National Center for Biotechnology Information and are listed in Supplementary File S1; Table S1. Each genome was subjected to RepeatMasker [6] (RepeatMasker-Open-4.0) analysis using a custom library, as reported in [3]. This library consisted of the original 62 Platy-1 subfamilies reported in [1], the two owl-monkey-derived subfamilies previously reported in [2], and *Alu* subfamily consensus sequences from RepBase [7]. Each RepeatMasker output was analyzed for full-length Platy-1 insertions, measured by having a start position no more than 4 bp from the 5′ start of the Platy-1 consensus sequence (positions 0 to 4) and an end position of ≥103. These full-length elements were then analyzed for the number of insertions for each Platy-1 subfamily. A flanking sequence of ±500 bp was added to the genome coordinates to generate fasta files to compare to the other twelve genomes by using a locally installed version of BLAT [8].

### 2.2. Interspersed Repeat Genome Content

Following the initial RepeatMasker analyses described above, the consensus sequences for Platy-1 element subfamilies were incorporated into a larger, more comprehensive RepeatMasker library and the thirteen genomes (Supplementary File S1; Table S1) were reanalyzed for overall interspersed repeat content. These analyses were used to determine the repeat landscape for each genome, including the distance to the nearest neighboring repeat. The ‘calcDivergenceFromAlign.pl’ and ‘createRepeatLanscape.pl’ RepeatMasker utilities were used to generate sequence divergence plots. Bedtools v2.29.2 [9] was used to analyze the number of Platy-1 insertions within 50 bp of other repeat types, such as *Alu*, LINE1 (L1), LTR and DNA transposons. The purpose was to determine the Platy-1 mobilization dynamics, with regard to target-primed reverse transcription [5] integration mechanisms, that are possibly unique to tamarins and to identify correlations that could be related to these expansions.

### 2.3. Platy-1 Nested Integration Within Existing Alu or L1

To calculate the number of Platy-1 elements that integrated directly into an existing *Alu* or L1 for each of the thirteen genomes, a targeted RepeatMasker run was performed. The library for these analyses consisted of consensus sequences for the Platy-1, L1, and *Alu* subfamilies (Supplementary File S2), and the query sequences for each run included all the full-length Platy-1 elements with a sufficient flanking sequence (fasta files) for each of the thirteen genomes. RepeatMasker output ‘ID’ numbers were used to identify incidences of intra-*Alu* or intra-L1 nested Platy-1 repeats. The purpose was to determine if Platy-1 elements were achieving any of the following: (1) mobilizing in conjunction with young L1 (i.e., hitching a ride); (2) neutralizing full-length L1 replication; or (3) none of the above.

### 2.4. COSEG Analyses of Lineage-Specific Subfamilies from AloPal_v1

Of the new genomes analyzed in this study, only one possessed a sufficient number of lineage-specific insertions to attempt a Platy-1 subfamily reconstruction, AloPal_v1: the mantled howler monkey, *A. palliata*. A combination of cross_match using default parameters (http://www.phrap.org/phredphrapconsed.html (accessed on 19 November 2025)) and COSEG (www.repeatmasker.org/COSEGDownload.html; accessed on 19 November 2025) analyses were completed to evaluate if any of the lineage-specific Platy-1 SINEs from the howler monkey genome constituted new subfamilies. Platy-1 elements were aligned to the Platy-1-4a subfamily consensus sequence and exact matches were eliminated. New COSEG-derived subfamilies were added to the local library, which was previously used for WGS analyses (combined library is available as Supplementary File S3) and RepeatMasker was performed again to determine new subfamily assignments and to calculate improved RepeatMasker output metrics such as Smith–Waterman score (SW) and percent divergence scores (% div) to accurately reflect Platy-1 activity in light of the newly discovered subfamilies.

### 2.5. Neighbor Joining Tree of Platy-1 Subfamilies

To visualize the placement of lineage-specific Platy-1 elements in Bolivian titi, as well as the newly discovered mantled-howler-monkey-derived Platy-1 subfamilies, in the context of those previously reported, Neighbor Joining trees [10] were generated using MAFFT version 7 [11], using the default parameters. Each tree was exported in the Newick format and the output was visualized by using FigTree v1.4.4. (http://tree.bio.ed.ac.uk/software/figtree/; accessed on 19 November 2025) and imported into PowerPoint for annotation.

### 2.6. Lineage-Specific vs. Shared Platy-1 Elements

Construction of the above-mentioned Platy-1 fasta files for each genome provided the opportunity to utilize these novel SINEs for platyrrhine phylogenetics by recording ‘presence/absence’ patterns across taxa. Each set of full-length Platy-1 MEIs with sufficient flanking sequence was subjected to BLAT [8] comparisons, as described previously [3]. Each genome was searched for either unique or shared Platy-1 insertions by looking for specific gap sizes between the input sequences and each of the target genomes. A custom python script, “inDepthSpecCheck1_platy-1” (available at https://github.com/t-beck; accessed on 19 November 2025), was used to computationally detect an ~85 bp gap (absence of the Platy-1 element) compared to other genomes. Ambiguous calls were resolved by manual inspection of sequence alignments in BioEdit (version 7.2.5) [12]. Shared elements (insertion present at the same genomic position) were assigned a genotype value of “1”, while absence of a particular element was given a “0” code and “?” was used to indicate missing values. ‘Specificity’ categories across all thirteen species included fixed-present (‘FP’—shared by all platyrrhine genomes studied), lineage-specific (‘LS’—unique to only one genome), polymorphic (‘Poly’—variable presence/absence patterns among studied taxa) or missing values (‘MV’—instances in which a particular locus had a “?” genotype) for seven or more of the thirteen taxa. ‘Specificity’ groups reported previously in [3] were *Saguinus*-specific (‘Sag’—shared by both *Saguinus* species but absent from marmoset, *Aotus* and cebids) or callitrichid-specific (‘Call’—shared by the three callitrichids while absent from *Aotus* and cebids). Within the ‘Poly’ designation are sub-categories that are indicative of being restricted to a certain platyrrhine family or subfamily. ‘Pith’ (present only in family Pitheciidae species—saki and titi); ‘Pith and Atelidae’ (present only in Pitheciidae and Atelidae species); ‘Atelidae-Ceb-Aot-Call’ (present across families Atelidae, Cebidae, Aotidae and Callithrichidae; absent only from Pitheciidae); ‘Atelidae’ (present only in family Atelidae; shared by the mantled howler monkey and the two spider monkeys); ‘Ateles’ (restricted to the two spider monkeys); ‘Ceb’ (restricted to traditional cebids—including Callithrichidae and Aotidae—while being absent from Pitheciidae and Atelidae families); ‘Cap’ (restricted to the three capuchins); ‘Saimiri and Cap’ (present only in the squirrel monkey and capuchins); or ‘Other’ (described in the text). These genotype distributions were used to infer phylogenetic relationships among taxa, compared to [13].

## 3. Results

### 3.1. Full-Length Platy-1 Elements

**Figure 1:**
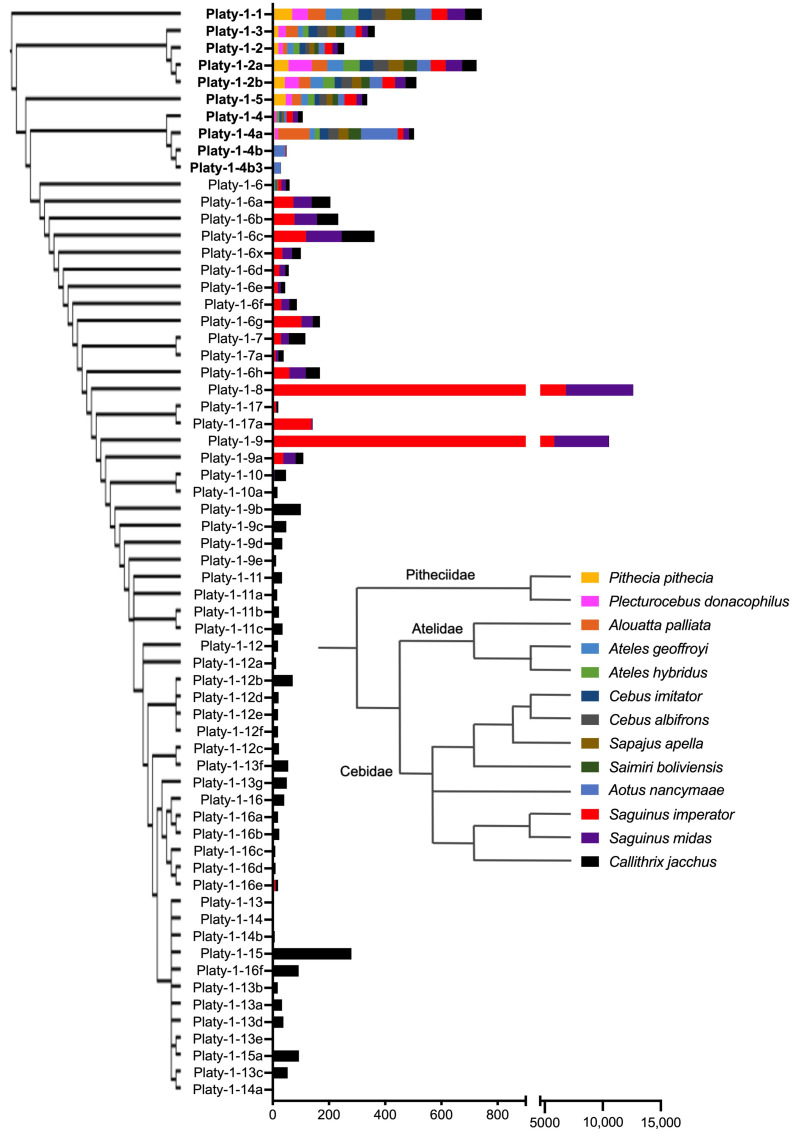
Platy-1 subfamily distribution among the analyzed genomes. The original 62 Platy-1 subfamilies reported in [1] are listed oldest to youngest, top to bottom, along with two subfamilies found in *A. nancymaae* (medium blue; Platy-1-4b and Platy-1-4b3) from [2]. The cladogram to the left of the stacked bar graph illustrates the subfamily relationships (adapted from [1]). The insert cladogram shows the generally accepted relationships among the studied taxa. The *X*-axis shows the number of Platy-1 elements per species, using a split scale to accommodate the several thousand Platy-1 MEIs found in tamarins, *S. imperator* (red) and *S. midas* (purple). The separation of bolded and non-bolded subfamily names on the y-axis shows the striking delineation of subfamilies unique to marmosets (younger than 1-9; *C. jacchus* (black) and illustrates that subfamilies younger than 1-6 are restricted to callithrichids (marmosets and tamarins). Among non-callithrichids, Platy-1 activity derives from older subfamilies.

RepeatMasker analyses, using a custom Platy-1 library [3] across thirteen platyrrhine taxa provide conclusive evidence that callithrichids, and *Saguinus* tamarins in particular, are indeed unique among platyrrhines with regard to their high level of Platy-1 mobilization. This answers a key question that emerged from our previously published investigation related to *Saguinus* tamarins [3]. Platy-1 content distributed by the subfamily for each of the thirteen platyrrhine genomes is shown in Supplementary File S1; Table S2 and is summarized in Figure 1.

The analyzed species are listed top to bottom in evolutionary order, as reported by Perelman et al. (2011) [13]. *S. imperator* (red) and *S. midas* (purple) each have several thousand copies from subfamilies 1-8 and 1-9 (split scale), as reported in [3], and both have a total copy number of Platy-1 MEIs that far exceed that of any other studied taxa. The number of elements from older subfamilies, Platy-1-1 through 1-6, are generally consistent across all taxa, with the exception of individual minor expansions in the Bolivian titi from subfamily Platy-1-2a (pink) and in the mantled howler monkey from Platy-1-4a (orange highlight; similar to the owl monkey, *A. nancymaae* in medium blue). Of the 62 subfamilies initially discovered in the common marmoset [1] (black), those younger than Platy-1-9a are essentially absent from the other twelve genomes, including the two tamarins. The Platyrrhine families Pitheciidae, Atelidae, Cebidae and Aotidae (all non-callithrichids) possess negligible numbers of Platy-1 elements that derived after the Platy-1-6 subfamilies, except for scattered singletons. From these analyses, only the owl monkey, tamarins and marmoset have elements with 0% sequence divergence (Supplementary File S1; Table S2: peach highlight), which is indicative of young insertions with ongoing proliferation.

**Figure 2:**
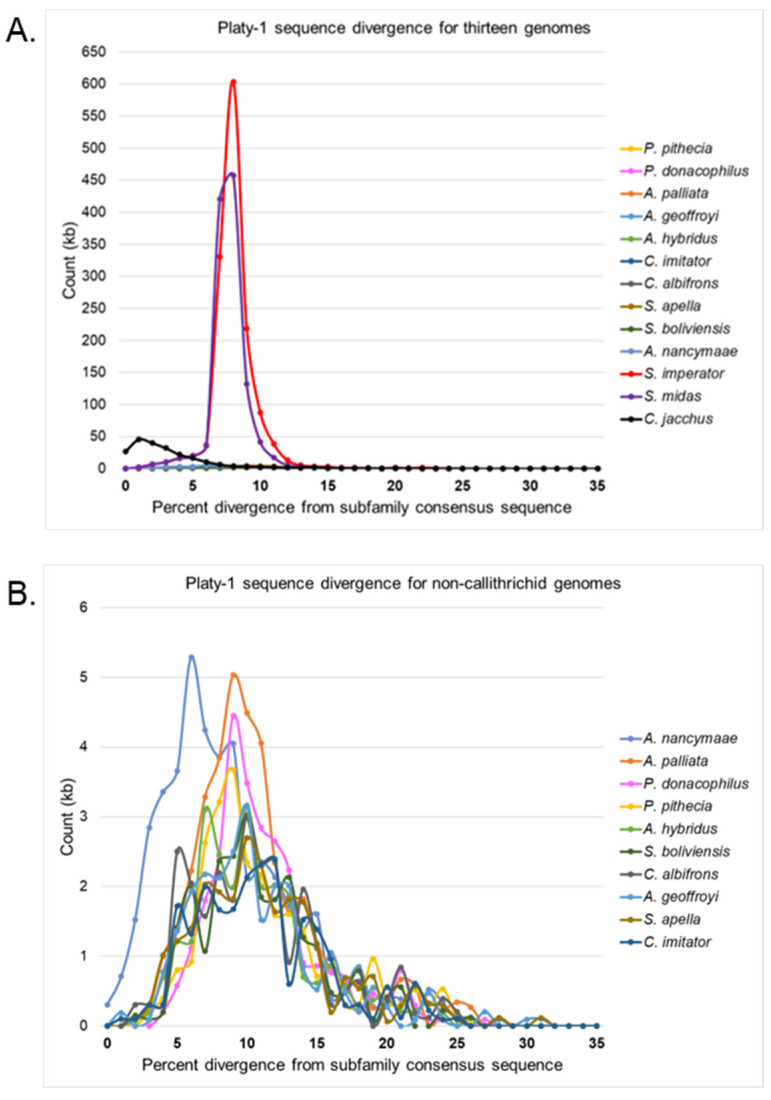
Platy-1 sequence divergence by genome content for (**A**). thirteen platyrrhine taxa and (**B**). ten non-callithrichids only with the species legend sorted by highest peak value to lowest. The percent divergence is shown on the *X*-axis and full-length Platy-1 element count in kilobase pairs (kb) is shown on the *Y*-axis for each species.

The genome contents of full-length Platy-1 elements, based on the percent divergence from the original subfamily consensus sequences, are illustrated for each of these thirteen platyrrhine taxa in Figure 2A. The tamarins *S. imperator* (red) and *S. midas* (purple) are strikingly different compared to all other species. Each tamarin has extensive Platy-1 content at about 5–10% sequence divergence from the original subfamily consensus sequences (primarily Platy-1-8 and 1-9), whereas the Platy-1 content in the marmoset (*C. jacchus*; black) has very low sequence divergence (≤5%), as expected given that these subfamilies were first discovered in the common marmoset [1]. These data are consistent with the overall copy number of Platy-1 elements in these callithrichid genomes (Figure 1; Supplementary File S1; Table S2) [1,3] and are indicative of ongoing Platy-1 element propagation in these lineages. The ten non-callithrichid genomes are not detectable on this scale and therefore are illustrated separately in Figure 2B. The owl monkey, *A. nancymaae* (medium blue); howler monkey, *A. palliata* (orange); and Bolivian titi, *P. donacophilus* (pink) each have notably higher peaks than the other genomes on this scale, which is consistent with having recent lineage-specific expansions of Platy-1 elements. The remaining platyrrhine species each have a small amount of their Platy-1 element genome content with 5–15% divergence, while most trend older with higher divergence.

The complete Platy-1 RepeatMasker output for the tamarins and marmoset was reported previously in [3] and is available for the ten non-callithrichid genomes from this study in Supplementary File S1; Table S3.

### 3.2. Platy-1 Amplification Dynamics and MEI Genomic Landscape

**Figure 3:**
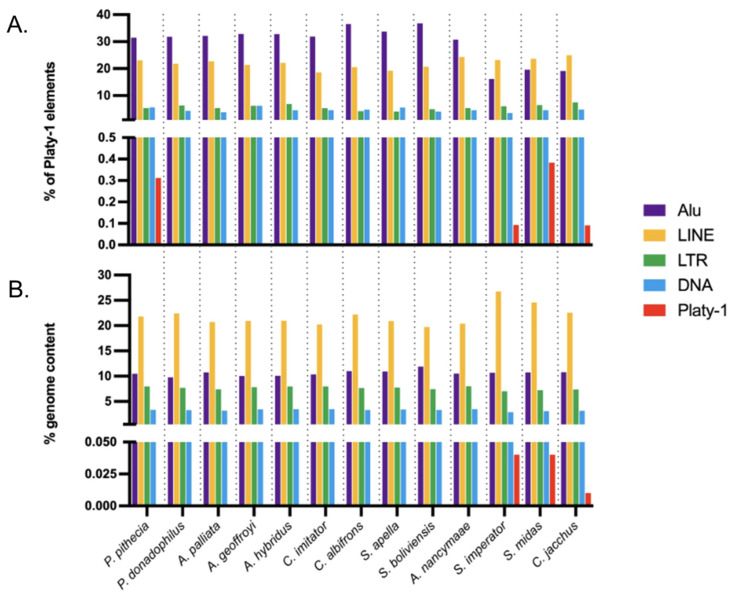
Genomic landscape of interspersed repeat content. (**A**). The percentage (%) of Platy-1 insertions located within 50 bp of another repeat. A total of 30–40% of Platy-1 elements in Pitheciidae, Atelidae, Cebidae and Aotidae (*X*-axis: left to right, all non-callithrichids) are located within 50 bp of an *Alu* element (purple), whereas this value is only 15–20% among tamarins, *S. imperator* and *S. midas*, and marmoset, *C. jacchus*. The % of Platy-1 elements within 50 bp of another Platy-1 (red) is illustrated in the bottom panel using an adjusted scale. (**B**). Total repeat content as a percentage of the masked genome. Top panel: LINE (L1) content (yellow) appears higher in callithrichids, especially in tamarins *S. imperator* and *S. midas*, than in other platyrrhine species. Bottom panel: Adjusted *Y*-axis scale to illustrate Platy-1 content. Dash lines extending from the X-axis visually separate species.

Thus far, RepeatMasker analyses in this study have focused on identifying Platy-1 elements from each genome. However, the stark differences between callithrichids and other platyrrhine taxa, with regard to Platy-1 mobilization, prompted investigation of the possible relationships between Platy-1 amplification dynamics and the surrounding genomic landscape of repeats. Platy-1 elements are known to propagate via TPRT and thus compete for the same LINE (L1) mobilization machinery that *Alu* elements do to achieve successful replication and genomic integration. Therefore, the extensive Platy-1 radiation observed in tamarins could be related to the density of L1, *Alu*, or other MEIs in close proximity to Platy-1 elements. To determine the overall interspersed repeat content, each genome was reanalyzed by using a comprehensive RepeatMasker library, which was comparable to the online version but with the addition of traditional Platy-1 consensus sequences (Supplementary File S1; Table S4). The MEI genomic landscape was analyzed to determine the density of repeat elements and the ‘nearest neighbor’ to Platy-1 elements across each genome. Figure 3A illustrates the percentage (%) of Platy-1 elements in each genome that are located within 50 bp of another repetitive element: an *Alu* (purple), a LINE (L1) (yellow), an LTR (green), a DNA transposon (blue), or another Platy-1 element (red—bottom panel). Among the Pitheciidae, Atelidae, Cebidae and Aotidae taxa, 30–40% of Platy-1 elements are juxtaposed within 50 bp of an *Alu* insertion. In contrast, only 15–20% of Platy-1 elements in Callithrichidae species are in such close proximity to an *Alu* element (Figure 3A top panel). These data are reported as percentages due to the drastic differences in actual copy number. The genomic density among the LTR and DNA transposons proximal to Platy-1 elements appears similar across the thirteen taxa. The *Y*-axis scale is adjusted in the bottom panel to illustrate the % of Platy-1 insertions within 50 bp of another Platy-1 element (red). *P. pithecia*, the white-faced saki, is the only non-callithrichid species that appears to have some adjacent Platy-1 clustering.

A markedly different density of *Alu* elements in proximity to Platy-1 insertions between callithrichids and all other platyrrhine species might infer that *Alu* amplification rates are possibly lower in callithrichids due to the vast expansion of Platy-1 MEIs competing for similar integration sites. To test this hypothesis, the RepeatMasker output for each genome was analyzed for total repeat content, as a percentage of the overall masked genome (Figure 3B). The overall genomic content of *Alu* elements is not lower in callithrichids and is actually quite similar across all taxa, as are the LTR and DNA transposon content (Figure 3B; Supplementary File S1; Table S4). However, these analyses reveal a notably higher LINE (L1) content (yellow) in the three callithrichids (top panel), particularly in tamarins (mean = 25.65%) compared to non-callithrichids (mean = 21.03%) (Supplementary File S1; Table S4b): a finding consistent with that of Ceraulo et al. (2021) [14], who reported a massive enrichment of LINE-1 elements in tamarins. Platy-1 repeat content, as a percentage of the entire masked genome, is only 0.04% in tamarins and 0.01% in marmoset (Figure 3B bottom panel; Supplementary File S1; Table S4e). These results indicate that the observed differences in Platy-1 to *Alu* density are not caused by quantitative differences in the number of *Alu* insertions, which are consistently plentiful in all studied taxa, but rather represent differences in the genomic landscape of young Platy-1 integration sites and L1 density between callithrichids and other platyrrhine species.

**Figure 4:**
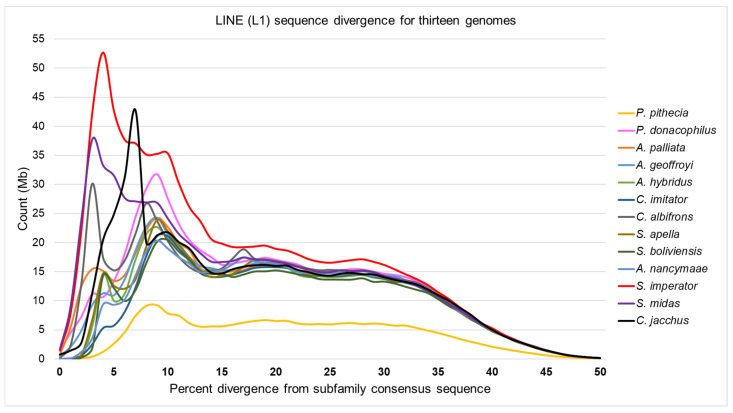
LINE (L1) percent sequence divergence is shown on the *X*-axis and genome content in Megabase pairs (Mb) is shown on the *Y*-axis for each of thirteen platyrrhine species. Tamarins *S. imperator* (red) and *S. midas* (purple) each have peaks of high genomic L1 content with ~2–3% divergence from subfamily consensus sequence. Marmoset (*C. jacchus*; black) has 43 Mb of L1 content with ~5% sequence divergence.

The observation that the L1 content appears greater among the tamarins and marmoset, as compared to other platyrrhine genomes, while Platy-1 copy numbers are also higher in these Callithrichidae lineages, might imply a cause-and-effect relationship. It is known that Platy-1 mobilization via TPRT requires the enzymatic machinery of L1. This prompted an analysis of L1 sequence divergence across taxa, in which a lower % divergence score indicates younger, potentially active elements. These L1 divergence plots are shown in Figure 4. The two tamarins, *S. imperator* (red) and *S. midas* (purple), each have large peaks at ~2–3% sequence divergence, which is indicative of a high content of relatively young L1 insertions. The marmoset, *C. jacchus* (black), also has a prominent low percent divergence peak at ~5%, with these three callithrichid genomes having the largest volume of recently integrated L1 content, compared to the other ten platyrrhines. Most other non-callithrichids have their L1 content peak at ~8% sequence divergence and trend higher (older) thereafter. Interestingly, the mantled howler monkey, *A. palliata* (orange), has a small (15 Mb) peak at about 3% divergence and the Bolivian titi, *P. donacophilus* (pink), has a 30 Mb peak at about 9% divergence, being somewhat consistent with their limited lineage-specific Platy-1 activity, as noted in Figure 1 and Supplementary File S1, Table S2. In contrast, evidence that is not supportive of an L1 to Platy-1 correlation is as follows: (1) the white-fronted capuchin, *C. albifrons* (gray), displays a pronounced 30 Mb peak at about 3% divergence, with no corresponding Platy-1 expansion and (2) the owl monkey, *A. nancymaae* (medium blue), has a very low young L1 content (9 Mb peak at 4% divergence, lower than other lineages) yet has two young lineage-specific Platy-1 subfamilies [2]. The raw data used to construct Figure 4 is available in Supplementary File S1, Table S5.

### 3.3. Platy-1 Nested Integration Within Existing Alu or L1

**Figure 5:**
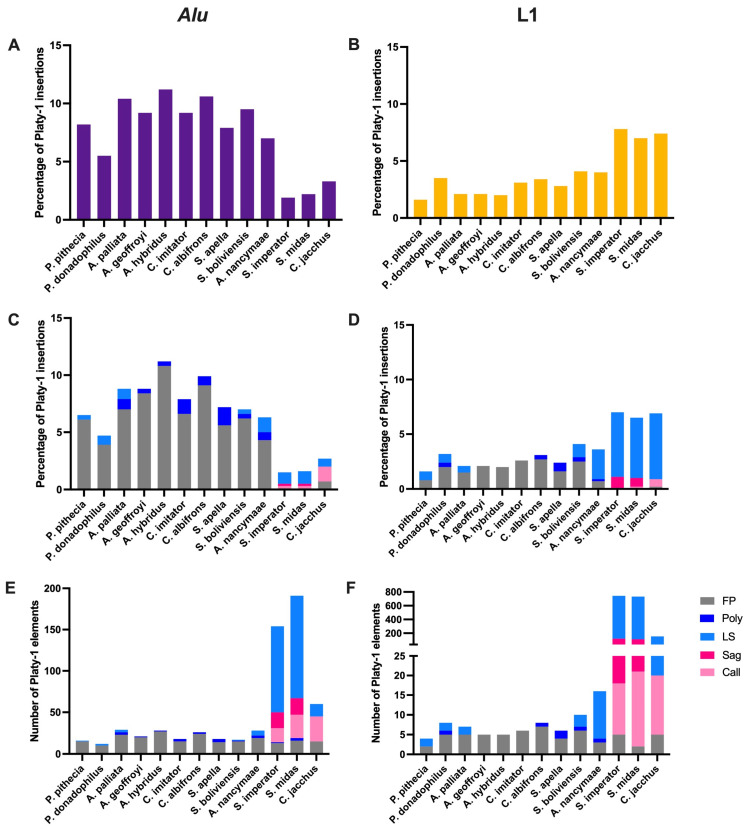
Full-length Platy-1 elements that inserted into an existing *Alu* (**left** panels) or L1 (**right** panels) upon TPRT integration into each of thirteen platyrrhine genomes. *Alu*: (**A**) Percentage of Platy-1 insertions per genome, with (**C**) describing the same dataset but separated by age category: FP—fixed present; poly—polymorphic among various taxa; Call—shared by and restricted to the three callitrichids; Sag—shared by and restricted to the two *Saguinus* tamarins; or LS—lineage-specific, restricted to that species. (**E**) Describes the data as actual numbers rather than as a percentage. L1: (**B**) Percentage of Play-1 insertions per genome, (**D**) split by age category, and (**F**) total number of intra-L1 Platy-1 insertions per genome, using a split scale on the *Y*-axis to accommodate hundreds within callitrichids.

The ‘nearest neighbor’ analyses above seemed to favor *Alu* proximity in non-callithrichids and greater overall L1 content in callithrichids. Concurrent evidence showing that the L1 content in callithrichids is generally younger (Figure 4), while these taxa also have an abundance of recently integrated Platy-1 MEIs, fueled speculation that perhaps Platy-1 elements are acting in concert with L1. Perhaps an L1-rich environment is providing a ‘safe haven’ for these small SINEs, a form of protection that facilitates their unimpeded proliferation in tamarins. Alternatively, perhaps the higher L1 content in callithrichids is deleterious to the host, and TPRT-derived Platy-1 MEIs are disrupting the L1 integrity, effectively neutralizing them. To test these hypotheses, a targeted RepeatMasker run was implemented to calculate the number of Platy-1 elements that integrated directly into an existing *Alu* or L1. A custom library consisting of Platy-1, L1, *Alu* and associated consensus sequences (Supplementary File S2) was used, with a query of full-length Platy-1 elements with sufficient flanking sequence (fasta files) for each of the thirteen genomes. The previous ‘nearest neighbor’ analyses counted these as two flanking proximal insertions, rather than residing inside another repeat. The complete results of this analysis for each of thirteen genomes are available in Supplementary File S4 and are summarized in Figure 5.

The overall percentage of full-length Platy-1 elements that inserted directly into an existing *Alu* or L1 upon TPRT integration is generally consistent across taxa, and ranges from about nine to fourteen percent. However, for the ten non-callithrichids, those intra-repeat integration events, or nested repeats, are dominated by insertions into *Alu* elements (Figure 5A), whereas the reverse has occurred for the tamarins and marmoset, where the vast majority of intra-repeat Platy-1 elements reside inside L1 elements (Figure 5B). These genome totals are divided into age-related integration categories in Figure 5C for inside an *Alu* element and Figure 5D for inside an L1, illustrating that older Platy-1 elements that have reached fixation (fixed present—‘FP’ shown in gray) generally landed within *Alu* elements during intra-repeat integration. This is particularly true for non-callithrichids. In contrast, the youngest Platy-1 insertions (lineage specific—‘LS’ shown in light blue), were overwhelmingly inserted into an existing L1 element when intra-repeat integration occurred. This is most striking for the three callithrichids but is also evident in the owl monkey (*A. nancymaae*) (Figure 5D). The actual number of Platy-1 elements with intra-repeat integration is shown in Figure 5E for *Alu* and Figure 5F for L1. The two tamarins, *S. imperator* and *S. midas*, each possess hundreds of Platy-1 elements integrated within an existing L1 (Figure 5F), requiring a split scale on the *Y*-axis to fully illustrate them. The marmoset (*C. jacchus*) also has nearly all LS intra-repeat Platy-1 elements within L1 (Figure 5F), as opposed to within an *Alu* (Figure 5E). Although the instances of intra-repeat TPRT integration are dominated by integration into existing *Alu* elements, especially for the oldest fixed-present (FP) Platy-1 elements, the youngest insertions (LS) have tended to land within L1, although to a far lesser extent than what is taking place in the tamarins. The vast majority of the *S. imperator* and *S. midas* lineage-specific Platy-1 elements that have integrated within an existing L1 element have precise TSDs and belong to the youngest *Saguinus*-specific subfamily Platy-1-8c_*Saguinus* (Supplementary File S1; Figure S1).

To assess the possible functional implications of intra-L1 Platy-1 integration, the RepeatMasker output (Supplementary File S4) was analyzed for the L1 subfamily and Platy-1 location within the L1 consensus sequence. Although the intra-L1 Platy-1 elements are themselves very young, the L1 host elements appear quite old, with many being from L1 ‘M’ subfamilies (meaning mammalian). The Platy-1 elements are generally located at the 3′ end of the L1 consensus sequences, within a range of positions ~3 kb to 6 kb. The L1s appear to all be 5′ truncated and most have >10% sequence divergence scores, meaning that they have age-related sequence decay. Therefore, Platy-1 integration is not disrupting L1 replication because the host elements are neither full-length, nor young. They are also not ‘hitching-a-ride,’ as these L1s are clearly not active. The youngest intra-L1 Platy-1 elements have precise TSDs, confirming their mobilization via TPRT, and have integrated into AT-rich regions. Among the *S. imperator* intra-L1, n = 3 have A-tails that are >100 bp in length and another 14 have A-tails that are 51–96 bp long and all are members of the youngest 1-8c_*Saguinus* subfamily, have TSDs and endonuclease cleavage sites. However, the closest TTTT termination signal is 22 bp downstream of the 3′ TSD, while most do not have any termination signal or have one much further away (Supplementary File S5). Therefore, these intra-L1 Platy-1 loci are unlikely to be active source elements themselves, despite being very young and TPRT-derived. Members of other *Saguinus*-specific Platy-1 subfamilies (prior to 1-8c) that are located within an L1 have A-tails < 25 bp, with most being < 20 bp (Supplementary File S5). Therefore, they are also unlikely to be active source elements from this location, although that cannot be completely ruled out. The inverse proportion of intra-*Alu* and intra-L1 Platy-1 insertions between non-callithrichids and callithrichids seems to be a consequence of genomic landscape differences in opportunistic landing sites, rather than due to a functional mobilization role.

### 3.4. Lineage-Specific Platy-1 Expansions in Bolivian Titi and Mantled Howler Monkey

**Figure 6:**
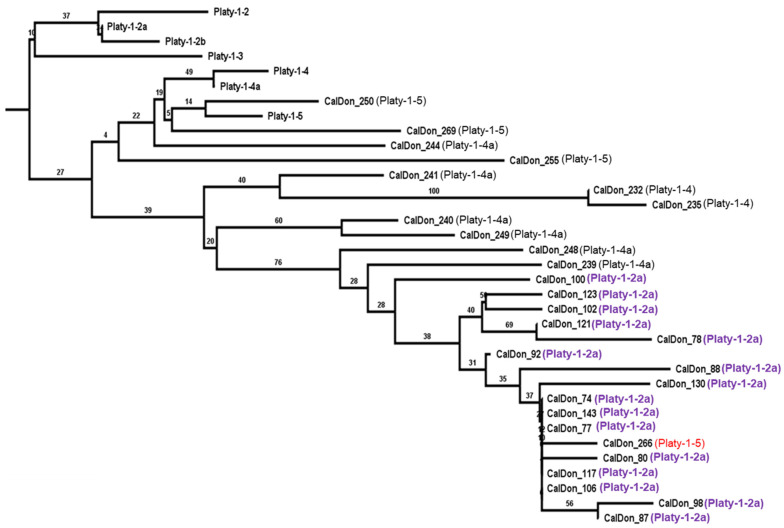
Neighbor joining tree [10] generated using MAFFT version 7 [11] of lineage-specific Platy-1 elements from the CalDon_v1 genome of Bolivian titi. Conventional Platy-1 subfamilies 1-2 to 1-5 (black) are shown along with n = 28 lineage-specific CalDon loci, 16 of which appear to be derived from a new subfamily that emerged after Platy-1-2a (purple). Their placement on this neighbor joining tree indicates that this lineage-specific mini-expansion likely originated from 1-4a to 1-5 (example in red) elements that remained viable following early platyrrhine evolution. The RepeatMasker subfamily designation for each LS insertion is shown in parentheses. The numbers on the branches are bootstrap values.

The initial RepeatMasker output for the thirteen genomes analyzed in this study indicated that two previously uncharacterized platyrrhine species might possess recent lineage-specific Platy-1 activity. The Bolivian titi, *P. donacophilus*, a member of family Pitheciidae, showed an increase in the number of Platy-1-2a insertions, and the mantled howler monkey, *A. palliata*, a member of family Atelidae, showed signs of activity from Platy-1-4a (Figure 1; Supplementary File S1; Table S2). Although somewhat tangential to the primary focus of this study, their discovery is noteworthy. Each was analyzed for lineage-specific (LS) insertions and possible young subfamilies. From the CalDon_v1 genome of *P. donacophilus*, n = 28 LS insertions were identified, with sixteen of those being generally designated as Platy-1-2a-derived (Figure 6). The neighbor joining tree includes the conventional Platy-1 subfamilies for Platy-1-2 to 1-5, along with the 28 LS insertions, shown by their ‘CalDon’ locus number (Supplementary File S1 Table S3). The RepeatMasker subfamily designation for each LS insertion is shown in parentheses. The sixteen LS insertions identified as Platy-1-2a (shown in purple) do not branch directly from the 1-2a consensus node but instead appear to be a new subfamily derived from the ancestral branch of the 1-4a and 1-5 lineage. Locus CalDon_266 might be miscalled as a Platy-1-5 element (red font), based on its placement on the tree among the ‘1-2a’ lineage-specific nodes (purple font). The numbers on the branches are bootstrap values (generally low support). The sequence alignments of these Bolivian titi lineage-specific Platy-1 elements support the possible sprouting of two very small ‘stealth’ driver [15] derived subfamilies (Supplementary File S1; Figure S2), each with an insufficient copy number to be amenable to COSEG subfamily analyses.

From the AloPal_v1 genome of the mantled howler monkey, *A. palliata*, n = 73 LS insertions were identified, with n = 64 being classified as 1-4a by RepeatMasker, n = 2 each as 1-2a, 1-4 and 1-5 and n = 1 each as 1-1, 1-2b and 1-3. Sixteen additional AloPal_v1 Platy-1 elements were determined to be shared by all three Atelidae species in this study (genera *Alouatta* and *Ateles*). These sixteen elements derived from subfamilies 1-4a (n = 7) and 1-5 (n = 9), indicating that each of these subfamilies had active source elements during the evolutionary time frame of the Atelidae radiation, although with limited propagation (Supplementary File S1; Table S6). COSEG analyses were performed using a combined fasta file containing the 73 LS sequences and compared against the Platy-1-4a consensus sequence. COSEG generated two new AloPal_v1 Platy-1 subfamilies, designated as sf0 and sf1. The newly generated consensus sequences for sf0 and sf1 were added to the custom RepeatMasker library (combined library is available as Supplementary File S3) and a targeted RepeatMasker run was performed for the 73 LS and sixteen Atelidae-specific Platy-1 elements to determine their potential new subfamily assignments and to calculate the improved output metrics. Of the 73 LS AloPal_v1 insertions, 68 were re-assigned to one of the two newly discovered subfamilies with improved Smith–Waterman (SW) and percent divergence (% div) scores, including all 64 of the original 1-4a loci. Of the sixteen Atelidae shared loci, eleven retained their original subfamily designation, including all nine 1-5 and two of the 1-4a elements. However, five of the 1-4a insertions were reassigned to new subfamilies (n = 4 to sf1, n = 1 to sf0), suggesting that sf1 is older than sf0 (Supplementary File S1; Table S6a). These new subfamily assignments resulted in improved output metrics for SW and % div scores by an average of 80.9 ± 33.9 and 2.3 ± 1.3, respectively, for sf1. Improved metrics were even greater for the younger sf0 subfamily, with SW up by 175.6 ± 27.7 and % div improved by 6.3 ± 1.3 points (Supplementary File S1; Table S6b). Sequence alignments for the combined LS and Atelidae-specific insertions, as compared to conventional Platy-1 subfamilies, support the accumulation of diagnostic nucleotide substitutions, ranging from Platy-1-4a to sf1, sf0 and 1-5, confirming that sf1 is older than sf0 (Supplementary File S1; Figure S3).

**Figure 7:**
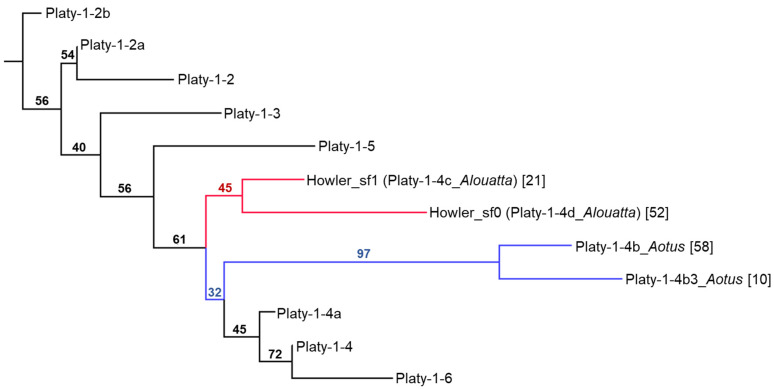
Neighbor joining tree [10] generated using MAFFT version 7 [11], illustrating the placement of two new howler monkey Platy-1 subfamilies (red lines) compared to conventional subfamilies Platy-1-2 to 1-6 (black lines) and the two *Aotus* subfamilies (blue lines) reported in [2]. Numbers on branches are bootstrap values. Numbers in brackets following subfamily names are the number of subfamily members. The *Alouatta*-specific subfamilies derived between 1-4a and 5 and emerged independently from *Aotus* subfamilies that also derived between 1-4a and 5.

These two novel Platy-1 subfamilies from AloPal_v1 are both derived from the ancestral Platy-1-4a subfamily, mirroring those previously identified in the owl monkey [2]. COSEG data were used in conjunction with sequence alignments to determine if these Platy-1-4a subfamily nodes, that were characterized from two separate genera (*Aotus* and *Alouatta*), represented coinciding evolutionary events, or if they occurred independently. The results conclusively show that the Platy-1-4a expansion in the mantled howler monkey, *A. palliata*, is different from the Platy-1-4b and 4b3 expansion that emerged in the *Aotus* lineage and that they likely originated from different 1-4a source elements and occurred independently (Supplementary File S1; Figure S4). A neighbor joining tree [10] illustrates the branch placement of the new howler monkey subfamilies, along with the *Aotus* subfamilies 4b and 4b3, as well as conventional Platy-1 subfamilies for Platy-1-2 to 1-6 (Figure 7). Because the new howler monkey subfamilies were discovered after the *Aotus* subfamilies were reported, and are independent branches, they were assigned new subfamily names (Howler_sf1 was renamed Platy-1-4c_*Alouatta)* and Howler_sf0 was renamed Platy-1-4d_*Alouatta*), using standardized nomenclature for MEIs [16] based on their consensus sequence and time of discovery.

This indicates that Platy-1 subfamilies 1-4a and 1-5 maintained active stealth drivers [15] that gave rise to small independent radiations in *Alouatta* and *Aotus* separately. The lineage-specific insertions that were previously reported in genera *Saimiri* and *Cebus* were all Platy-1-4a or a tiny number of 1-5 [2] but only a handful had ≤2% divergence scores, and none were polymorphic on a multi-sample DNA panel (n = 32 *Saimiri* and n = 14 *Cebus* individuals, respectively) [2]. Therefore, *Saimiri* and *Cebus*, both of platyrrhine family Cebidae, lost the active driver elements in these subfamilies, while genera *Alouatta*, of the Atelidae family, and *Aotus* (Aotidae) retained active source elements that have generated recent daughter copies independently in each lineage.

Of the 68 LS AloPal_v1 loci assigned to new subfamilies, n = 30 have ≤2% divergence scores from the new subfamily consensus sequence, with n = 2 having 0%, n = 12 having 1%, n = 5 having 1.9% and n = 11 having 2% (Supplementary File S1; Table S6c). These low-percentage divergence scores indicate that they are relatively young insertions and could possibly be polymorphic for presence/absence among *Alouatta* species, like those discovered in the *Aotus* lineage (31 insertions were polymorphic among 119 analyzed by PCR across 23 *Aotus* individuals: a 26% polymorphism rate) [2]. Therefore, we designed potential oligonucleotide primers for PCR for these young insertions to assess their utility for *Alouatta* phylogeny (in preparation).

### 3.5. Shared Platy-1 Insertions and Platyrrhine Phylogenetics

Ascertainment of full-length Platy-1 elements (with 500 bp of flanking sequence) from thirteen platyrrhine genomes for this study presented an ideal opportunity to utilize these novel SINEs for computational phylogenetics. Each was “genotyped”, as compared to the other twelve taxa, for insertion presence/absence patterns, using in-house ‘SpecCheck’ (described in Section 2). The results for all Platy-1 insertions across thirteen species are shown in Supplementary File S1; Table S7. A summary table for each of the three Callithrichidae species (marmoset and tamarins) was reported previously [3]. Table 1 summarizes the values for each genotype category for the ten non-callithrichid genomes.

A list of lineage-specific (LS) insertions from Table 1 is shown with their RepeatMasker subfamily for each species in Supplementary File S1; Table S8. Within the polymorphic (Poly) classification (Supplementary File S1; Table S9) are sub-categories that are indicative of being restricted to a certain Platyrrhine family, subfamily or ‘Other’ (Table 1). These sub-categories (defined in Section 2) generally support the traditional platyrrhine branching order of Pitheciidae/(Atelidae/Cebidae—including Aotidae and Callithrichidae), while also showing the independent radiation of Platy-1 MEIs among related species. For example, the three capuchins (‘Cap’) share about 18 to 25 insertions that are absent from all other species, along with an additional n = 4 that is only shared with the squirrel monkey (*Saimiri*), which is consistent with the close relationship between the platyrrhine subfamilies Saimiriinae and Cebinae of the currently recognized Cebidae family. Also, several shared insertions (7 to 11) support the grouping of traditional Cebidae (‘Ceb’), which previously had included the owl monkey, tamarins and marmoset, but are now considered a three-family clade of Cebidae, Aotidae and Callithrichidae. The two spider monkeys (genus *Ateles*) share five or six Platy-1 insertions that are absent from all other species, whereas 14–16 shared insertions group the members of family Atelidae together. Even the most basal family of platyrrhines, Pitheciidae, share at least four relatively recent insertions that are absent from other taxa. A sequence alignment of (n = 10) lineage-specific Platy-1 insertions from *P. pithecia* (white-faced saki) and an additional n = 4 shared with *P. donacophilus* (Bolivian titi) is shown in Supplementary File S1; Figure S5. This alignment illustrates that Platy-1 activity that is specific to the Pitheciidae family of platyrrhines is derived from subfamilies Platy-1-4, 1-4a and 1-5. These lineage-specific insertions display relatively young-looking A-tails of up to 40 bp in length, suggesting that these are recent integrations that occurred long after the divergence of the Pitheciidae family from other platyrrhines.

An explanation of those assigned to “Other” is available in Supplementary File S1: Table S10. These anomalies typically involved either a genomic deletion, in which a particular species lacked additional sequence besides the absence of the Platy-1 element, or were indicative of incomplete lineage sorting (ILS) among rapidly segregating taxa. However, there was a single notable exception: a Platy-1-4a insertion polymorphism that is shared among Cebidae and Callithrichidae species while having a precise pre-integration site among Pitheciidae, Atelidae and Aotidae (Supplementary File S1; Figure S6). Based upon the ‘identity by decent’ principle of SINE insertions, in which the absence of a Platy-1 element at a given locus represents the ancestral state, this finding suggests that Aotidae (owl monkey) is phylogenetically basal to both Cebidae and Callithrichidae, as opposed to being placed between Cebidae and Callithrichidae, as proposed by [13]. The branching of *Aotus* after family Atelidae, but prior to *Cebus*-*Saimiri* and the group of callitrichines, is consistent with the multispecies phylogenetic tree reported in [17], although this exact order was considered inconclusive, leaving the position of *Aotus* as the only remaining “enigma” of the platyrrhine phylogenetic puzzle. The fact that callitrichines have exponentially more Platy-1 SINE insertions compared to other platyrrhines, and the bulk of which are derived from younger subfamilies that originated after Platy-1-6, provides strong support that the expansion of Platy-1 elements in the tamarins and marmoset occurred after their divergence from *Aotus* and cebids. No insertion polymorphisms were identified that supported an alternative topology. However, a single locus is clearly insufficient evidence for phylogenetic conclusions. This locus could also represent a case of ILS or perhaps a precise parallel insertion in *S. imperator* and *S. midas*, based on subtle sequence differences. However, the sequence of the third callithrichid, the marmoset (*C. jacchus*), is nearly an exact match with cebids (Supplementary File S1; Figure S6), linking Callithrichidae and Cebidae to the exclusion of Aotidae.

## 4. Discussion

Analyses of Platy-1 retroposons and other MEI content from thirteen different species, representing all currently recognized platyrrhine families, illustrate that the extensive Platy-1 expansion observed in *Saguinus* tamarins [3] is unique not only due to their strikingly large copy number, but also in the source of their proliferation. Each analyzed genome from this study had evidence of lineage-specific Platy-1 amplification on some level. Among the families Pitheciidae, Atelidae, Aotidae and Cebidae, recent Platy-1 activity has originated from the older subfamilies Platy-1-4, 1-4a and 1-5 that radiated about 20 MYA [1,2]. The term “stealth driver” in MEI evolution [15] implies that there was a long period of semi-quiescence before an ancient active driver began to propagate daughter elements. However, the entire radiation time among the aforementioned taxa is only ~1–2 MY, having occurred about 19–20 MYA [13]. This means that the source elements responsible for the recent lineage-specific mobilization of young Platy-1 insertions observed in the Bolivian titi monkey (Pitheciidae), mantled howler monkey (Atelidae) and owl monkey (Aotidae) [2] are all derived from separate source drivers, each being 19–20 million years old. These 1-4 to 1-5 ancestral subfamilies retained active ‘sleeper’ elements that have yielded a recent progeny that is unique to each of these platyrrhine lineages. Some of these Platy-1 elements are relatively young, most notably in genus *Alouatta* from this study, and *Aotus* as reported in [2], with each harboring two new lineage-specific subfamilies that derived from 1-4a.

However, the bulk of the recently integrated Platy-1 SINEs has occurred in family Callithrichidae, with an unprecedented eruption observed in two *Saguinus* tamarins thus far. This implies that their mobilization might be augmented by additional mechanistic pathways compared to other platyrrhine families [18], not only because of the vast explosion of very young elements in the tamarins, possessing over six times more than was found in the marmoset, and over 7500 unique copies in each of two different *Saguinus* species, but also in the source node leading to their amplification burst. In contrast to non-callithrichids, where the universal norm is now shown to be residual activity from older subfamilies, all six of the newly discovered *Saguinus* Platy-1 subfamilies were derived from Platy-1-8 to 1-9 source nodes [3]. These are much younger branches that are further down the subfamily tree [1] and ones that now appear to be restricted to Callithrichidae. All other platyrrhines in this study had essentially zero copies of Platy-1 elements from any subfamilies younger than 1-6. These data are strikingly robust, showing the stark differences between callithrichids and non-callithrichids with regard to Platy-1 amplification dynamics.

Thus, factors that can influence TPRT mobilization via L1 were investigated. One key factor might be differences in the MEI genomic landscape, showing a larger number of young L1 elements in conjunction with Platy-1 activity among callithrichid species. Full-length autonomous L1 elements encode two proteins, ORF1p and ORF2p, which are required for their retrotransposition [19,20], whereas TPRT-derived mobilization of non-autonomous MEIs, such as *Alu*, SVA and Platy-1, relies primarily on ORF2 from L1 elements [21]. Although having ORF1 is advantageous, only the endonuclease and reverse transcriptase enzymatic functions of ORF2 are strictly required for TPRT [21]. Furthermore, the ORF2 sequence of L1 can sustain multiple mutations within the endonuclease domain without impairing the enzymatic function or facilitation of TPRT [22]. We observed hundreds of instances in which young Platy-1 elements were inserted into an existing L1 element. However, all the L1s were 5′ truncated and quite old. Although ORF2 can remain functional within 5′ truncated L1 elements, the severely advanced age-related decay of these particular L1s makes it exceedingly unlikely that they were providing the endonuclease and reverse transcriptase directly to their Platy-1 residents [23]. Rather than disrupting L1 mobility or using them as a ‘launching pad’ for proliferation, these Platy-1 integrations seem to be finding suitable endonuclease cleavage sites for a ‘soft landing’ within an L1 sequence that is already tolerated by the host genome. Evidence of ‘Repeat-in-Repeats’ has been reported previously for Tf2 retrotransposons [24] as well as for *Alu* elements [25]. Transposable elements tend to possess AT-rich regions such that the presence of each insertion thereby increases the availability of the endonuclease cleavage sites available for target priming of another one (typically 5′-TTTT/AA-3′), as reported in [19,26]. This scenario provides a mechanism by which to balance their own ‘selfish’ survival with that of the host genome. The identification of the specific L1 elements that are responsible for Platy-1 mobilization is not a straightforward undertaking and is certainly beyond the scope of this study.

Although this study suggests a correlation between Platy-1 and L1 activity, possibly augmenting the TPRT mechanistic processes among tamarins, identification of any specific catalysts remains elusive. Active lineage-specific L1 subfamilies have been identified in *Saguinus*, while genus *Ateles* has relatively low levels of recent L1 evolution [27]. This seems to support the theory that the availability of young L1 elements is driving Platy-1 expansion. However, the *Saimiri* lineage of the squirrel monkey also has strong evidence of young L1 amplification, evolving into several species-specific subfamilies [27], with no corresponding Platy-1 proliferation [2]. Although the overall *Saimiri* L1 content was not shown to be higher in this study, the simple availability of young L1 subfamilies does not seem to automatically lead to Platy-1 mobilization without the influence of specific host factors. Ceraulo et al. (2021) [14] used fluorescence in situ hybridization (FISH) analyses to demonstrate that tamarins have a massive accumulation of L1 in centromeres compared to other mammals. This L1 enrichment reportedly occurred prior to the common ancestor of all tamarins and is associated with the inter-chromosomal rearrangements and evolution of the tamarin karyotype [14]. It is possible that genomic restructuring could allow these small SINEs to evade host defenses and proliferate in certain altered environments. It is also worth noting that the vast majority of the Platy-1 eruption observed in tamarins was generated from the youngest subfamily, 1-8c_*Saguinus*, which has a distinct 9 bp indel in the consensus sequence, the last 8 bp of which represents a duplication of the immediate 5′ proximal consensus sequence of the 1-8 subfamily [3]. This unique variant might play a role in its unprecedented, and seemingly unimpeded, replication within *Saguinus* tamarins.

Thus far, only two *Saguinus* and one *Callithrix* species within the family Callithrichidae have been analyzed for genome content of Platy-1 SINEs. It is important to remember that Platy-1 elements were first discovered in the common marmoset (*C. jaccus*) [1] and as such, all subsequent studies have used that initial subfamily tree as the default benchmark for comparison, regardless of the evolutionary branching order of the studied taxa. The fact that *Callithrix* is a relatively distal branch of marmosets, radiating after tamarins but before genus *Mico*, means that our approach to investigating Platy-1 evolution is taking place in an often backwards or a ‘zig-zag’ fashion. It is possible that some (or many) of the original 62 Platy-1 subfamilies are themselves stealth drivers in other callithrichid species, or inversely, are descendants from ancestral subfamilies that are yet to be discovered in earlier radiating taxa. Therefore, the next logical step is to analyze as many tamarins (and marmosets) as possible to delineate evolutionary branching patterns associated with low, medium and high levels of Platy-1 proliferation. From these future analyses, a more narrowed focus might emerge regarding specific differences in genomic structural variation related to Platy-1 chromosomal distribution patterns that potentially generate enhanced mobility. By looking at a broad spectrum of Callithrichidae species in subsequent comparisons, a specific timeline can be established of when and where the Platy-1 amplification burst in tamarins occurred. This strategy could elucidate potential catalysts, versus the residual stealth sources struggling for survival that seem to be the norm among non-callitrichines.

During the timeframe in which the extensive analyses for this project were being undertaken, many more WGS for primates of the Americas have been released, including two chromosome-level assemblies for tamarins, *Saguinus oedipus* (Cotton-top tamarin, GCA_031835072.1 submitted by The Ohio State University) and *Leontopithecus rosalia* (Golden lion tamarin, GCA_028533165.1 submitted by the DNA Zoo, Baylor College of Medicine). Many more are scaffold-level assemblies that have been generated by a relatively small number of high-throughput sequencing centers, such as the Institute of Evolutionary Biology in Barcelona, Spain (IBE, CSIC-Universitat Pompeu Fabra), who have released another seven tamarin assemblies, at least, in recent months. These should be analyzed for Platy-1 content, followed by as many additional marmoset species as possible, to more accurately identify the timeframe of Platy-1 expansions and potential catalysts that are unique to tamarins.

The extensive Platy-1 expansion observed thus far in *S. imperator* and *S. midas* is not only strikingly unique among platyrrhines but could represent one of the largest independent bursts of MEI activity ever reported in primates: not in the percentage of the occupied genome, as with much larger L1 elements, but certainly in terms of having such a high copy number that has been generated over a very short evolutionary time period. The *Saguinus* radiation spans roughly 15 MY, with the branch leading to *S. imperator* diverging from the mystax group of mustached tamarins approximately 8 MYA, while *S. midas* split from the *midas-bicolor* clade about 5 MYA [28]. Thus, *S. imperator* and *S. midas* each accumulated over seven thousand Platy-1-8c_*Saguinus* MEIs independently in less than ~5–8 MY. Analysis of additional tamarin species should elucidate the ‘when, where and why’ answers to these questions.

Each primate lineage develops a unique set of MEIs. Transposable element bursts of genomic activity are reported to be the driving forces for evolutionary change [29,30,31]. Sudden explosions of lineage-specific MEIs are often deleterious to the host genome and are eliminated from the population rather quickly, or they can lead to rapid restructuring of the genome and evolutionary adaptation. Although this study does not pinpoint any specific catalysts that triggered the massive tamarin Platy-1 expansion, the genomes appear to tolerate the intrusion thus far. These Platy-1 elements do not seem to have a functional role in driving evolutionary change: rather, they are an inadvertent byproduct of it. Perhaps the interactive dynamics of rapid structural variation and the expansion of L1 content results in genomic instability that allows Platy-1 propagation to thrive via L1 mediated integration without extensive damage to the host. Perhaps these very small SINEs are more easily tolerated by the genome, compared to the highly disruptive propagation of full-length L1 elements, and are somehow related to the many callithrichidae-unique genomic features, such as adaptive phyletic dwarfism. The emergence of family Callithrichidae, with genus *Saguinus* being the first to diverge, occurred about 15 MYA [13]. Following *Saguinus*, the traditional evolutionary divergence order is *Leontopithecus* (the lion tamarins), *Callimico* (Goeldi’s marmoset), *Callithrix*, *Mico* (marmosets) and *Cebuella* (pygmy marmoset), gradually trending from larger-bodied to smaller-bodied primates as proposed by the phyletic dwarfism hypothesis [13]. Future investigations of other tamarin and marmoset species may unveil possible relationships between Platy-1 mobilization and speciation events.

Compilation of Platy-1 elements and their associated genomic flanking sequences for this study facilitated multiple tangents of investigation that were unrelated to the massive expansion in tamarins. Recent activity of lineage-specific Platy-1 subfamilies in the Bolivian titi and mantled howler monkey helped to illuminate our overall understanding of Platy-1 amplification dynamics from older subfamilies among non-callithrichids. Potential howler monkey insertion polymorphisms for genus *Alouatta* are now being investigated (in preparation). In addition, these datasets furnished a comprehensive collection of genotypes and presence/absence patterns of shared versus unique insertions among these thirteen taxa. While not necessarily suitable for independent publication, the first broad application of Platy-1 SINEs for platyrrhine phylogenetics could not be ignored. Although a Platy-1-based phylogeny using PAUP was not performed, due to the limited number of informative (i.e., polymorphic) loci, these ‘essentially homoplasy free’ presence/absence patterns demonstrated that each lineage and all platyrrhine families harbor a unique accumulation of Platy-1 MEIs, and that their distribution by subfamily can inform phylogeny. Conventional phylogenetic groups were well supported, such as Pitheciidae–Atelidae–Cebidae (including Aotidae and Callithrichidae), with several shared Platy-1 elements supporting each of these three platyrrhine families and their branching order. Genus-level support for *Ateles*, *Saguinus* and *Cebus* was also confirmed, along with grouping together of the squirrel monkey (Saimiriinae) and capuchins (Cebinae) to form the currently recognized Cebidae family. Among the three-family clade of Cebidae, Aotidae and Callithrichidae, family Callithrichidae was the last to emerge. Segregating Platy-1 MEIs provided clear confirmation of this, leaving only the evolutionary order of Aotidae (the owl monkey) versus Cebidae unresolved using these data. A single Platy-1-4a locus placed Aotidae basal within the three-family clade, before Cebidae, as opposed to between Cebidae and Callithrichidae. This study, and others [1,2], have demonstrated that the 1-4a subfamily was segregating at the time of the Aotidae–Cebidae radiation, lending credibility to this finding. While no Platy-1 loci specifically placed the cebid branch basal instead, a single locus certainly cannot be considered to be a concrete phylogenetic placement. However, this locus was much easier to identify amidst a background of relatively few polymorphic Platy-1 MEIs, as compared to a previous study using *Alu* alignments in which each of the studied taxa was so inundated with a high copy number of *Alu* SINEs that ‘near-parallel-insertions’ and ILS muddled any definitive conclusions [32].

The relatively small number of Platy-1 elements found among the studied cebid taxa more closely resembles the content found in spider monkeys, genus *Ateles*, whereas *Aotus* has evidence of recent Platy-1 activity, which perhaps makes it appear more similar to Callithrichidae than Cebidae. However, the *Aotus* activity derives from 1-4a, not 1-8 and beyond, so the discovery of the 1-4a subfamily radiation in *Alouatta* seems to dispel the theory that *Aotus* must be linked to callithrichids, based in part on having recent Platy-1 activity. These Platy-1 data still have evidence of ILS despite their very slow rate of mobilization during early platyrrhine evolution, where ‘identity by descent’ should theoretically be easier to track due to the presence of fewer insertions. Primate ILS is known to be pervasive, especially among platyrrhines and specifically at this node. As reported in [33], 29 of 49 primate phylogenetic branches showed evidence of ILS, with scores ranging from 5% and 64%. The platyrrhine lineage exhibited some of the highest ILS scores: 59.4% at the emergence of platyrrhines, reaching the highest value reported in that study of 64.4% at the branch leading to *Aotus* and Callitrichidae [33]. In a different study [34], Kuderna and colleagues used ~3500 probes of ultraconserved elements (UCEs) to generate a maximum likelihood genome-wide phylogeny across 233 primate genomes. They reported strong support for all interfamilial relationships, each having a posterior probability equal to 1.0 (100% support), except for the position of family Aotidae. A posterior probability score of 0.56 placed owl monkeys as a sister to Callitrichidae, rather than Cebidae. The fact that primate phylogeny could be resolved with near-100% certainty at all other nodes in that comprehensive investigation, while leaving the order of Cebidae and Aotidae at a nearly 50/50 chance, indicates that their emergence was almost simultaneous.

## 5. Conclusions

Platy-1 amplification dynamics in Callithrichidae, specifically in *Saguinus* tamarins, are unique among platyrrhines, proliferating an unprecedented number of copies from very young source nodes. *Saguinus* tamarins have had the largest Platy-1 expansion of any platyrrhine genus reported to date. Other non-callithrichid platyrrhine genera have limited recent Platy-1 activity derived from more ancient subfamilies. This study provides the first evidence of recent Platy-1 mobilization for an Atelidae species, with the characterization of two new Platy-1 subfamilies in the mantled howler monkey, *A. palliata*. Platy-1 SINEs are emerging as another useful resource of ‘identical by descent’ insertion polymorphisms for the study of platyrrhine phylogeny, such as is underway for genus *Alouatta*. However, the confident placement of family Aotidae remains unresolved.

Although this study did not conclusively identify any potential catalysts leading to the massive expansion of Platy-1 MEIs in tamarins, advancements in sequencing platform technologies and the rapid generation of WGSs will greatly benefit future studies.

## Figures and Tables

**Table 1:** Distribution of presence/absence patterns for full-length (FL) Platy-1 elements with flanking sequence (w/fasta). FP (fixed-present); LS (lineage-specific); MV (missing values); Poly (polymorphic); Pith (Pitheciidae); Ceb (traditional cebids); Aot (Aotidae); and Cap (capuchins).

Platyrrhine Family	Pitheciidae	Pitheciidae	Atelidae	Atelidae	Atelidae	Cebidae	Cebidae	Cebidae	Cebidae	Aotidae
Common Name	White-Faced Saki	Bolivian titi	Mantled howler monkey	Black-handed spider monkey	Brown spider monkey	Panamanian white-faced capuchin	White-fronted capuchin	Tufted capuchin	Bolivian squirrel monkey	Owl monkey
Genome	PitPit_v1	CalDon_v1	AloPal_v1	AteGeo_v1	ORGONE_01	Cebus_imitator1.0	CebAlb_v1	GSC_monkey_1.0	sBol_2.1	Anan_2.0
FP	211	207	206	208	222	181	211	204	186	223
LS	10	28	73	2	2	0	0	0	23	168
MV	18	13	29	6	3	16	11	9	17	41
Poly	6	7	19	23	22	31	42	39	16	14
Total FL w/fasta	245	255	327	239	249	228	264	252	242	446
Polymorphic loci (Poly row above) separated into sub-categories.										
Pith	4	5	0	0	0	0	0	0	0	0
Pith and Atelidae	0	1	0	0	0	0	0	0	0	0
Atelidae-Ceb-Aot-Call	0	0	2	2	1	1	1	1	2	3
Atelidae	0	0	16	14	16	0	0	0	0	0
Ateles	0	0	0	6	5	0	0	0	0	0
Ceb	0	0	0	0	0	7	10	11	8	9
Cap	0	0	0	0	0	18	25	21	0	0
Saimiri and Cap	0	0	0	0	0	4	4	4	4	0
Other	2	1	1	1	0	1	2	2	2	2
Total Poly	6	7	19	23	22	31	42	39	16	14

## Data Availability

The algorithms used in this study are available on GitHub (https://github.com/t-beck; accessed on 19 November 2025). The Supplementary Data Files are available in the online version of this paper and through the Batzer Lab website under publications, https://biosci-batzerlab.biology.lsu.edu/, accessed on 19 November 2025.

## Supplementary Materials

The following supporting information can be downloaded at: https://www.mdpi.com/article/10.3390/genes17010100/s1, Supplementary File S1 is an Excel file with worksheets for supplementary Tables S1–S10 and Figures S1–S6. Supplementary File S2 is a *.fa file for a custom RepeatMasker library, comprising consensus sequences for the Platy-1, L1 and *Alu* subfamilies. Supplementary File S3 is a *.fas file as a custom RepeatMasker library of Platy-1 subfamily consensus sequences, including two newly discovered from the howler monkey, as well as those discovered previously in the marmoset, owl monkey and tamarin [1,2,3]. Supplementary File S4 is an Excel file with worksheets for intra-*Alu* and intra-L1 integration of Platy-1 MEIs for each of the thirteen genomes. Supplementary File S5 is a PDF file illustrating TPRT characteristics for some tamarin intra-L1 Platy-1 insertions.

## Abbreviations

The following abbreviations are used in this manuscript:

SINE	short interspersed element
MEI	mobile element insertion
TPRT	target-primed reverse transcription
WGS	whole genome sequence
MYA	million years ago
