# Supplementary material for: Platy-1 SINEs from Thirteen Diverse Genomes Reveal Callithrichidae Unique Amplification, Recent Alouatta Mobilization and Insights into Platyrrhine Phylogenetics
Source: Genes (Basel). 2026 Jan 19;17(1):100. doi: 10.3390/genes17010100 (PMC12840619; doi:10.3390/genes17010100)
Supplement: Supplementary file 1 [file genes-17-00100-s001.zip › genes-4035299-supplementary/genes-4035299-Supplementary File S5.pdf]

Supplementary File S5

Examples of intra-L1 Platy-1 (grey highlight) integration via TPRT (TSDs are shown in red font); if a TTTT termination signal is present, it is shown in yellow highlight.

A. (N = 3) *S. imperator* intra-L1 Platy-1 elements with A-tails over 100 bp long
B. (N = 14) *S. imperator* intra-L1 Platy-1 elements with A-tails 51-96 bp long
C. *S. imperator* intra-L1 Platy-1 elements from other *Saguinus* specific subfamilies

A.

>SagImp_10886 Platy-1-8c_*Saguinus*, LS, inside L1PA17; Endo:GATT/GG
tctatCAAAGGATAGTTTTCAAAAAAGGGGTAAAATCATGACTTTCATACAtaagatataaaattgacacatgt
taaagattttatttaactCATTATTAATGAGGAAACCAGGAAGAGTTCAAGGAAGCACCAATTTATATGAAGT
GAAGGAACGTGGAACTAGATTGTGGATGCATATAAAACCGGCTTGCTACAGGGACTCACATGAACCTCCAGCAG
ACAACATAGTTATTAGTTATCTACAAATTATAGCAGTGCAAATATTTCATAGAGAGTAAAATGCTAGATACATT
ATAAGTAACTtcaacaaatcaacaagaaagaaatcaaataatcCCTTTAAAAAGTCGgtaaaagacatgaatag
acacttctcaaaagaagacatacgagtGGCCAACATATAAAAAAAAGTGCAACATAActtatcatcagagaaat
gcaaattaaaaacacagtgagataccatctcacaccaatcagaatggctactatcggggcccaaagtggctccg
gccggatgtcgtggcacttgcgaaggcgaggccaagagttcaaggccaaccaacctcagcaaccttacaagctc
ccaatgattggcaaaaaaaaaaaaaaaaaaaaaaaaaaaaaaaaaaaaaaaaaaaaaaaaaaaaaaaaaaaaaa
aaaaaaaaaaaaaaaaaaaaaaaaaaaaaaaaaaaaaaaaaaaaaaaaaaaaatcagaatggctactatcaaaa
agtcaagaaacaacagatactggcaaAGTAGTGGGGAgaagggaatgcttatatgctgtaagtaggaatataaa
ttacttcagcccctgtgaaaagcagtttggagatttctgaaaggactaaaaatagaactaccagcaatcccttt
acttagtgtatacccaaaggaaagtaaataattctaccaaaaagatgCCTGCACTCATATATTTATcacactat
tcataatagcaaagacatggaatcaacccaggtgcccatccACGTTGTGGCAGGTaatgtggataaagaaaata
tggcatgtacacatcatggaatactatgcagccacagaaaagaataaaatcatttcccCCAGCGACATAG

>SagImp_8721 Platy-1-8c_*Saguinus*, LS, inside L1M1; Endo: CTTT/AT
GACCAATTGGATCTAACagacatatataaaatgctgcaCCTAATAAtgacagaatacacatttttctcAAGTG
CACaggaacattcttcaggatagacTATGTGTTAGACTGTAATACAAGCCTTAATGAATTTAAAGGCACTGAAA
CATGCTAAGCATCCTTTCTGATCAAAATGTAATGAAACTAGAAGACTGGAAAATccacaaatatgtagaaatta
aacagTACACTATTAAACAACCAATAAGTTATATAAGAAATTataagagaaaactgaaaatatctTGAAtcaaa
tgaagataaaaataccACATAGCAAAACTTACAGAATGCAGTGAAAGTACTGCTAAGAGATAAGTTTGTTACTC
TAAaagcttaaatttaaaaaagaaaaaaaaattaaatcaattcTGTAACTTTACACTTTAAGGAACAAGAAGAG
GAGGAGCAAATTGAACTTAAATGTAGCAAAAAGTGGGAATAATAAAGATGcagcaggggcccaaagtggctccg
gccggatgtcgtggcgcttgcgaaggctaggccaagagttcaaggccaaccaacctcagcaaccttacaagctc
ccagtgattggcaaaaaaaaaaaaaaaaaaaaaaaaaaaaaaaaaaaaaaaaaaaaaaaaaaaaaaaaaaaaaa
aaaaaaaaaaaaaaaaaaaaaaaaaaaaaaaaaaaaaaaaaaaaaaaaaaaagatgcagcagagataaataaaa
tagagaacaaaaagtatagagaaaatcaacaaaaccatgAGTTGATTCTTCAACAggtcaacaaaattgacaaa
cctttaagCAAATTTACtaggagagaggacacaaataagagggaacacttcctaactcattctatgaggccagc
attatcctgacaccaaaaccagacaatGGCACTATAAGAGAAGAAAACTGCAGTTGAATATCTctatgaacatt
gatgcaaaaatcctcaagaaaatactggcaaactaacttcaacagcatattaaaaggatCATATACCATGACCA
AGGGAGATTTATTTCTAGAATACAAGAATGATTCAAGATGTGAAAAGCAATCAATGTAATATACCATAtt

>SagImp_10950 Platy-1-8c_*Saguinus*, LS, inside L1P3; Endo:TTTT/GA
caacagacgcaagactataaatgtaattcaccacataaacataagcaaaaacaaaaaccacatgattatctcaa
ttgatgcagagaaggcatttgacaaaattcaacagccctttatgctaaaaccctcaaaaatttggtattgat
ggaacacatctcaaagtaataaaagctatttatgacaaaccaatagccaatatcatacttaatgggcaaaaact
agaagcattctctttgaagtccggcactagacaagaatgccctctctcaccactcctattcaacatagtactgg
aagttctagctagagcaatcaggcaagacaaagaatcaaagtgtattcaaataggaaaggtggaagccaaattg
tctttatttgcagacgacatgatagtatacctcgaagacgccatcacctcagctcaaaagatcctgcaacttat
aagcaactttagcaaagtctcaggatataaaatcaatgttcaaaaatcacaagcaggggctcaaagtggctccg

gccggatattgtggcgcttgcgaaggtgaggccaagagttcaaggccaatcaacctcagaaaccttacaagctc
tcactgattggcaaaaaaaaaaaaaaaaaaaaaaaaaaaaaaaaaaaaaaaaaaaaaaaaaaaaaaaaaaaaaa
aaaaaaaaaaaaaaaaaaaaaaaaaaaaaaaaaaaaaaaaaaaaaaaaaaatcacaagcattcctatacaccaa
caacaaacttaaagagacccaaatcaagaaccaactgccattcacaattgctacaaagaagataaatacctgg
gaatacaactcagaagcaaagtaagggacctctttaagaaaaacaacaaaccactgctcaacgaaataagaaag
gacacaaacagatggagaaacattccatgttcgtggttaggaagaattagtatcatgaaaatggcaatactgcc
caaagtaatttacacaatcaatgctatccccatcaaactaccattgaatttcttcacagaactggaaaaaaaca
tcatgaatttcatatggaaccaaaaagagcccgcacagccaagtcaatcctaagcaaaagaacacagc

A. N = 14 *S. imperator* intra-L1 Platy-1 elements with A-tails 51-96 bp long

>SagImp_1379 Platy-1-8c_*Saguinus* in L1P1; -strand:rc; Endo: TTCT/AA
cccctcccctcccctccccttcccttcccttcctttcccttccttctctctctctttctctctttagtCTTACT
TTGtctgcagtggcgcgatcttagctcactgcgaactccacctcccaggctcaagcgattctcctacctctgct
tcctgagtagctgggactgcaggggaacaccaccatgcccagcttttctttttttaatttttcttagagacggg
gtttcactatgatggctaACATAGTCTCTAtttactgacctcgtgatccaccagcctcggcctcccaaagtgct
gggattacagccctttatgctaaaaactctcaataaactaggtattgatggaatgtatctcaaaataataaatg
ttatttatgaaaaacccaaagcccatataatactgaatgggcaaaaactggaaacattccctttgaaaactggc
acaagacaagggtgccctctctcaccactcctattcaacatagtattagaagttatgggggcccaaagtggctc
cggccggatgtcgtggcacttgcgaaggcgaggccaagagttcaaggccaaccaacctcaaaaaccttacaagc
tctcattgattggcaaaaaaaaaaaaaaaaaaaaaaaaaaaaaaaaaaaaaaaaaaaaaaaaaaaaaaaaaaaa
aaaaaaaaaaaaaaaaaaaaaaaaaaaaaaaaaaagaagttctggctagagaaatcaggcaagaaaaaaaaaa
aagatattcaattaggaaaagagaaagtcaaactgtctctatttgcagatgacataattgtatatttagaagat
cccattgtcttagcccaaaatctccttaagataatAAGCAAATTCaatgaagtctcaggatacaaaatcaatgt
gcaaaaatcacaagcatttctatacaacaataacagacaaatagagagccaaatcatgagcaaactcccattcg
cAATTGTttcaaggagaataaaatacctaggaatacaactatcaagggatgtgaaggacctcttcaaggagaat
tacaaaacactgttcaaggaaataagaaaagacacaaacagatggaaaaatatttccattctcatggttag

>SagImp_4056 Platy-1-8c_*Saguinus* inside L1P1; - strand:rc Endo:TTTT/CT
tgtgaaaatgcccatactgcccaaggtaatttattgattcaatgctatccccatcaagctaccaatgaccttct
tcacagaactggaaaaaaacacttcaaatttatatggaaccaaaagagagcgtGCATAACCAaaagaatcctaa
gcaaaaagaacaaagctgaaggtatTATGTtgcctgactttaaactatactacaaggctacagtaagcaaaaca
gcatggtactgttatcaaaacagagacatagaccaatggaacagaacagaggcctcagaagcaacaccacacat
ctacaatcatctaatctttgacaaacctgataaaaacaagcaatggggaaaggactctctgttgaataaatgat
gataggaaaactggctagcaatgttcagaaagcagaaactggaccccttcctgacatcttacactaaaattaac
tccagatagattaaagatttaaacataagacctaacaccagaaAAACCTTagaagaggggcccaaagtggctcc
ggccggatgtcgtggcacttgcgaaggcgaggccaagagttcaaggccaaccaacctcagcaacctcacaagct
ctcaatgattggcaaaaaaaaaaaaaaaaaaaaaaaaaaaaaaaaaaaaaaaaaaaaaaaaaaaaaaaaaaaaa
aaaaaaaaaaaaaaaaaaaaaaaaaaaaaccttagaagaaaacctaggcaaaatcattcaggacataggcatag
gcaaggacttcatgactaaaacaccaaaaacgatggcagcaaaagccaaaatagacaaatgggatctaattaaa
ctctagagcttctgtacagcaaaagaaacaatcattagaatgaactggcaacgaacagaatgggaaaaaagttc
ctgcaatctacccatctgacaaactgccaatatccagaatctacaaagaactaaaacaaatttacaagaataaa
acaaccaaacccattcaaaagtggatgaaggatatgaacagacacttttcaaaagaagatatatctgaggccaa
tgaacatatgagaaaatgcccatcatcactggtcattagagaaatgcaaattaaaaccacattgagatac

>SagImp_2013 Platy-1-8c_*Saguinus*, inside L1MA1, -strand:rc Endo: TTCT/AT
TTATGAAGGggtcaggggcagaatgatatggtttggttctatTTCCCTGTCCAAATCTTATCTCAAGTTGTAAT
TCCAACATGTTGGGGAAGGATCCTGGTtggattgaatcatgggagcacaCTTCCCCCTTGCTGCCCTTCTgtta
gtgagttctcaggagatctggttatTTGAAAGTGTATGACACTTCCCCCTTTGCagtctctctccctcctgcca
tCATATGAGATGTGTCTTTCTTCCTGTTcatcttttgccatgattgtatattgcctgaggcttccccagcctcA
GGCACTGTGTGTCAAttagacctcttttctttataaattacccagtctcacgtagttctttatagtagcctgaa
aatggactaatataaagACTATTATGCTAAGttaaacaagccaggcacagaaagacaaataacgTATGTTCTTA
CTTTTATGTGGAATCTGTAACAGTTAAACTCTAGATGTAGAGAATAGAACAAtagtggggcccaaagtggctct
agccggatgtcgtggcacttgcgaaggcgaggccaagagttcaaggccaaccaacctcagcaaccttacaagct

ctcaatgattggcaaaaaaaaaaaaaaaaaaaaaaaaaaaaaaaaaaaaaaaaaaaaaaaaaaaaaaaaaaaaa
aaaaaaaaaaaaaaaaaaaaacagaacaatagTTACCAGAGATTTAGGAGTGGGAGCAATGAAGAGATATTGGT
GAAAGTGTGCAATGTTTCTGTGAGgcagaagaaataaatggtaATTATTAAGGTGATGGGTATGAACTTGATTT
AGTCATTGCACATTGTATGCATTTATTATAGCATAAGTCTATACCCCGTAAATGTAAATTACTTAAATATGTCA
ATATTCAATATAGTATAATACACATACTGTTGTACCCAAGGGAGTCAAGGAATGCGCCACACGCTGAAATTGAG
TTATGAGTCTTTTATTGAGCCAGAGACAGAGTGGCTAACGCCGAAAATTCTTGTGGCCCCGAAGAATAAGTTAG
GTTGTCTTTTATACATTAGTTTTAACAGGGAGGGGAACCTAGCTGAAGTAAAGTTtttacaaaagcagag

>SagImp_1220 Platy-1-8c_*Saguinus*, inside L1M1, - strand:rc Endo:GCAT/GT
CAAACAGGGGGGCTGGGGGTGCAAATGCAGTGTACGAAGTACATTGCCTGCTAAACAACACAATACTCTTTGGA
GAAGcaaaagaacagaacagaatctaGAGTTTCTTTTTCGggggagatgggggtctcactattttgccgaggct
ggtctcaaactcctgagctgaagcaaccctcctgtctcagcctcctgagtagctaggactacaggcataccacC
ATGTCAGACTTGAATCTAGAGTTTCTACAACTTATTCATGATGTCCAGAATATATCcaaaattactccacatgt
aaaGAAGCAAAACACccatttcttaagaaaaaatcAGAATCATTTAAATAAAGGAATACTTAGGTATAAACTTT
ACAAAACATACATAGGATTTATatactgaaaattacaaaatgctcATGAAAAATTAAAGACCAtctaaattaat
aaagatattccatgttcatggaataGAAGACTCAATGTGGTAAACATGCTgattcaggggcccaaagtggctcc
ggccggatgtcgtggcgcttgtgaaggcgaggccaagagttcaaggccaaccaacctcagcaacctcacaagct
ctcatggattggcaaaaaaaaaaaaaaaaaaaaaaaaaaaaaaaaaaaaaaaaaaaaaaaaaaaaaaaaaaaaa
aaaaaaaaaaaaaaaaaaaatgctgattcAAAATGATCTGTAAGTTAAATGAGATTCCTATCCAAATTCTAATA
AGTTTCTTTTTATAAACATGAATAAGCTTATTTAAAAACTtatataaaaaggcaaaaaaaaaaagctgctaaaA
TAGTGAGAACAATATGGAAAGGAATAAAGTGGAAGGAACACCTGCTGATACTGGCGTTTACTTTATATAGGTAG
AGTCATCAAGATAGGGAAGTACTATAGAGGGAAAGAGACACATGGATCAATGGAACTGAAAAAAGAACCCAGAA
ACCCACACAAATATGCCTAAGtgaactttctttttcctcttgctcgttgctcaggctgcaacctctgcctccta
ggttcaagcagttctcctgcctcagcctcctgagtagctgggactacaggtatatgccaccacagctggc

>SagImp_2905 Platy-1-8c_*Saguinus*, inside L1M1, -strand:rc Endo:TTTT/AA
TTGTTGGATGACTAACATGTAATTTGAATATTTAAagatacaataatcaaaacagtgtggtttGGTATTAagat
agagaaaaagaagaatagaacagaatagagagttcaGAAATAGACTCAAACATATATATGGATAACTGGCATTG
AACAAAGGTTCAAAGGCATTTCCTTAGAGAAAGGATGGTCTTTTCCACAAATGGTGCTGAAACTAttgaatatc
catatgcaaaaactAAGCTTCTATCCATAATTGGTACCAAAAACAGAAGTTAGCTTAAAATGGATTATACACCT
AGATACAATACATTAAACATAAATctctagaataaaataaaagaaaatactttgtaaCCTTAGTCAAAAGACT
TAGATAAAACATCAAAATCCTAATCCATCAAAAAATGTATGGACCTTTGCAAAATGAAAAGCATTTGTTTTCTG
AGAGACTCTGTTATAAgaattcaaaaacaaagaaaatatttaaaaatcatatatctggggcccaaagtggctcc
ggccggatgtcgtggcgctcgcgaaggcgaggccaagagttcaaggccaaccaacctcagcaaccttacaagct
ctcattgattggcaaaaaaaaaaaaaaaaaaaaaaaaaaaaaaaaaaaaaaaaaaaaaaaaaaaaaaaaaaaaa
aaaaaaaaaaaaaaaaaatcatatatctgataaaggatttgTATTTACAATACATAAAGACATCTtaaactgaa
taataaaacaaCTCTACTTTTTTCAAAACTGGATTAAAAATTTGAACAGTTATTTTACCAAAGAGGATATAGTA
ATGGCAAATGGTCATAAAAAAATATGATCAATACAATTACAATGCGAAAATATGATATACCTAatagaatgact
aaaattaaaaatattttccacaaCAACTGTTAGTGAGGAAATGGGGGAACTATAAGTCACATACACTACTTGTG
AAAATGTGAAATTATACAACCATTTTGGGAAACAGTTTGAAACTTTATTAAGAggttaaaaatatatctatatc
acATATAGTTTCCTAAGATAAATgaaagtatatatttatacaaaatcTTGTATATTAGGGCCCATAGTAG

>SagImp_12928 Platy1-8c_*Saguinus*, LS, inside L1M1; Endo:TTAA/AA
cagaatacacattattttcctcaGCAGATGGATCAGGGTCATTCTGAAGAATAGAGCATAAGTTAGGTAACAAA
ACACGTCTTTAAACATTCAAAATTTGAAATGATATTAAGTATCTTTTCTGATGAGAATGATATAGatgtagaaa
ttaataacaaaacaaattttgGGAACTATAcgaatacatggaaattaaaccacATGCTCCTGattgaccagtgg
gtcaatgaagaaattaagaaggagaTTGacaaatttctcaaaaaaatgataatggaaacacagcataccaaaat
ttgtgggatgcagcaaaagcagtattaagaggaaagttcatagctaTAAGTGCCAAtatcaaagaataataaaa
aacttcAAATGTAAAAATCTAATGAGGTATCTTAACTGAATAAGTcaaagcaaaccaaacccaaaattagaaga
aaagaaataatgaagatcaaagcagaaatgaatgaaattaaaatttaaaatacagggtcccaaagtggctccg
gccggatgtcatggtgcttatgaaggcgaggccaagagttcaaagccaaccaacctcagcaaccttacaagctc
tcattgattggcaaaaaaaaaaaaaaaaaaaaaaaaaaaaaaaaaaaaaaaaaaaaaaaaaaaaaaaaaaaaaa
aaaaaaaaaaattaaaatacaaaaattagtgaaacAAAAAGTTGATTCTTTGGAAAGTTATATAAATTGACAAA
CCTTTGGtttgacagagagagagagagagagagggagagaggaagagatggTCCAATAGGAAATAATAAAGGac

acattacaactgatactgcagaaattcagataattagtggctactatgagcaattATACGCTAATAAATTGAAA
TATCTAGTAAAAAGAGACAAACTGACAGACACATACCACCTACCaaaattgaaccaggaagaaatccaaaCCCT
GAACAAACCAGAAAGAGTAACAAGATCAAAGCTATAATAAAATGTATCCCACTAAAGAAAAGCCAGAGACATGA
TGGCTTCACTTCTGAATTttatgaaacattaaaaatatatatcataccaatccttctcaaactattctga
>SagImp_4337 Platy-1-8c_*Saguinus*, LS, inside L1P3;-strand:rc; Endo:TTTT/GT
cacccaaaacaGAAGCACCtagttttataaaacaaattcttGAGCCTAAGACCTACACAGACACTTAGGCTCCT
gtacaataacagtgggagatttcaacatcccACTGTCAAGATTggacagatcaataagacagaaaattaacaag
gatattcaggactaaactcagctctggatcgTGTGGACCTAGTAgatgtctacagaactctctaccccaaatca
atagaatatacattcttctcagtgccacataaCACTTATTCTAAGATCAACCAAGTAATTGaattaaatcactc
ctcagcaaatgcaaaagaactgaaatcataacaatctctcagaccacagcgtCATCAAATTAAAACTtaggatt
aaaaaactcactcaaaaaccacacaatttcatggaaattgaacaacttgttccagaatgactcctgggtaaatg
aagaaattaaggcagaaataaagaagttctttgaaaccaacaaaaacaaaaagacagggcccaaagtggctccg
gccggatgtcgtggcgcttgcgaaggcgaggccaagagttcaaggccaaccaacctcagcaaccttacaatctc
ccaatgattgacaaaaaaaaaaaaaaaaaaaaaaaaaaaaaaaaaaaaaaaaaaaaaaaaaaaaaaaaaaaaaa
aaaaaaaaaagacaacctaccagagtctctgggataCAGCTCAAGTAgtgttaagaaggaaatttatagcacta
aatgcccacataagaaagtTAGAAACATCTCAAATCGACAACCTAAcctcacaactaaaagaactagagaggcA
AGAGTaaactaatccaaaagctagcaagagacaagaaataaccaagatcagacaAGAATTGAAGAAGCTAGAGA
CATAAaaatgctccaaaaaaaaaaatgatacatctaggagctagttttttgaaaaatattaacaaaatagataa
acccTAGCTACactaatagagaagaaaagagagaagaatcaaaccgacagtaaaatgttattaaatgagatatc
accactgaccccacagaaatacaaactcagAGAATACTATATAACCTCTATGCCAATAAACTAGaaagt

>SagImp_10709 Platy-1-8c_*Saguinus*, LS, inside L1MA2; Endo:GTTT/GT
ctgaCAAATTCACTAAAGtggcagaatacaaaattaatatacaaaattcaGTACTTTTTTTACAAGTACAATAA
ACTAGCTGAAAAATAAGttaagaaggcaatcccatttaaaatatctataaataataaatacctaggaataaatt
ttttaaagaagtttaaaactactacaaagaaaactataaaacaattataaaataaattagattCAAATGGAAGG
ATATCCTatgctcatgaattggaagaattaaaaCTGCTAAAGTGGtaatactatccaaagcaatctacaaactC
AGTACAATCCCTACCAATaacattatttacagaaatagaaCAAAAATTGTGAAATTTGTCTCAAGCTACAAAAT
ACCTCTAATAGCCCAAAAAATTATCAGCAAAAATAGAAAAGCTGGAATTATCACACTACCAGATcacaaaatat
actacaaagttgTAGtgaacaaaatggcaggatactagcataaaaacaaactaaaggggcccaaagtggctccg
gccggatgtcgtggcgcttgcgaaggcaaggccaagagttcaaggacaACCAACCTctgcaaccttacaagctc
ccaatgattggcaaaaaaaaaaaaaaaaaaaaaaaaaaaaaaaaaaaaaaaaaaaaaaaaaaaaaaaaaaaaaa
aaaaaaactaaaaatagaactaccatatgattcagcaatcctactactggtaatttatccaaaagaaaaaaata
tgtatcataTCAGAGAGACATCTACACCCTggtgtttattgaagcactattcacaatagtcaagatataaAATG
AACCTAGGTGTCCAgtaacagatgaatgaataaattgcaatgtatataccatggaataatattcagccataaaa
gaggaAACCATGATAGTCAAAGCAACATGGATGGATTAAACAAGAGGGAATATTAAAGgaaataagccagaaac
agaaagtGAAACATTCCACgttttcactcatatgtggaagctaaaggCAGTTAATCTCAAAAGGCAAAAAGTAA
AACAGAGTATACTAGGGTCTGGGAAGGGTATAATAAAATGAAGGGAGGGATAGGGACAACTTtgctaaaa

>SagImp_9359 Platy-1-8c_*Saguinus*, LS, inside L1PA16; Endo:GTTT/AG
acctcacatgagtcagaatgactattaataaaatgtcaagaaacaacagatgttggcaatggtgcagagaaaag
ggaatgcataTACACTGTGGGTGGGTATATAAATTACTTCAGTCACTATGAAAATCAGTTCagtgatttctcaa
aaaacttaaaatggaactaccattcaacccagcaatcctattactggatatatattcaaaagaaaatgaattgc
tctactaaaaagacacaagTATTCACATGTTCATCgtagcaccattcacaatagcaaagatgtggaatcaaccc
aggtgcccatcaatggcagATCAGATATACATCAGATATGTatacatcacagaatactacacagccgtataaaa
taatgaaattgtgtcctttgcatccacatggatggatctggaagccattatccgGACAaattaatgtaggaaca
gaaaaccaaatgctacatattctcacttataagtggaactAAACATTGGAtacttggggcccaaagtggctccg
gccggatgtcgtggcgcttgcgaaggcgaggccaagagttcaaggccaaccaacctcagcaaccttacaatctc
ccaatgattggcaaaaaaaaaaaaaaaaaaaaaaaaaaaaaaaaaaaaaaaaaaaaaaaaaaaaaaaaaaaaaa
aaaaacattggatACTCATGAACATAAATATGTCAAAAgaaactggggactactagaggagGGAGAAAATGAGA
GGGGATGGGTTGGAACATTATTGGGTACCATGTTCAGTACCTGGATGATAGGACTActcatactccaaacctca
gcatcatacaCTATTCCCaggtaacaaagctgcacatatactcctgaatctaaagttgagaaagagagaaaaag
taggcaaaggatattaatagacatttcttttcttttgacacAAAGCAGCTATTTATTAACATTCATAGAgactg
agtacagtggctcatgcctgcaattccagcgctttgagaggcatAGACGGAAggtcccttgagtccaggagttt
gagaccagccttggcaacatagggaaatcccatgtctattaaaaaatgtacaaaaattattagccaagca

>SagImp_9417 Platy-1-8c_*Saguinus*, LS, inside L1P3; Endo:CTTT/AA
ggaggCACTAGAATGAACTGTCAGGTTTCTGGCTTGGGCAACAAGGTAGGTACAGGTGCCATTTCCTTAGGAAG
GAAAGACACGGTGGCACCTGGAGTTGAGGAGCCTATTTCAACTGCAGTTCCACTGGAGAAGTAAGCCCAAAAGC
CCCAAGGCTTCACAGTCTGGAATGAATGTCTCTCCCCTGCATCTGAAATGGTACTAGTTATGTACctcagttct
ccttgaagaactGAGAATCTAGGCCTCGAGTCTTTTTCTGCATTCTGTGTTTCAGCCCAGTTGAGAAAGGTTTA
GAAAATCTAGGTCtataatgaaacagaatagagaactcaaccatcttatctttgacaaacttgacaaaaacaag
ccaagGTTGAAGGACtctaataaatggtgatgggagtGCTGGCTCGCCATATGCAAAAcattgaaactgaaccc
cttccttacaccacagacaaaaattaactcaagatggattaaagacttaaatgtaggggcccaaagtggctccg
gccggatgtcgtggcgcttgcgaaggcgaggccaagagttcaaggccaaccaacctcagcaccttacaagctc
tcaatgattggcaaaaaaaaaaaaaaaaaaaaaaaaaaaaaaaaaaaaaaaaaaaaaaaaaaaaaaaaaaaa
aagacttaaatgtaaaacccaaaactataaaaaccctagaagaaaatctagacaatatcattcaagacataggc
acagaaaaagatttcatgacaaaaacgccaaaaacaattgcaaaaaagcaaaaattgaccaaTGCgatctgatt
aaactaaagagattctgtacagcaaaataaagtatcatcagaatgaacaacctacagaatgggagaaaattttt
tgcaatacatccatctgacaaaagtctaatatccacaatctacaaagaacttaaacaagtttacaagaaaaaaa
tcaactccattaaaaagtgggtaaaggacatgaacagacacttcttaaaagaagacatacgtgcggccaataaa
catataaaaaaagctcaacataactgatcattagagaaatgcaaatcaaaaccacaatgagataccatct

>SagImp_8892 Platy-1-8c_*Saguinus*, LS, inside L1M1; Endo:TTTC/AT
TCCTTTCTGTGATAtctttttggaaaaagaaaaatatatatataatgatttattatttttaaaactatgagaAT
AACTGATATCAGTAAAGAGAGCAATGAAATAATTGCCCAGATCATCTAGATTAGTTTTAACTTTATTATTCAAT
TCATTCAGAATAAAAAGGGCagttttgactaaaataaataatcaggatAAAAATGATTTGGGCTAAGTTACTAA
GACACTCTCTTCTTCaatttctctctttgttttgcatttataatagaaaaatcttCACCTAATGAACATCAGGC
CAGgtcaaaaaatatttctattcaaTTATAGAGacattttaaggaaaaattactaggtttattcttaaatattt
tattctttttgatgctactgTGTAGTTGAAGACAGTTTGTTGTTAGCATATAAGTACTTGGAAATAAaatgaac
caaggaggtgaaaaatgtatacactgaaaactatgaCACTGATGAAATTAacacaggggcccaaagtggctccg
gccagatgtcgtggcgcttgcgaaggcgaggccaagagttcaaccaacctcagcaaccttacaatctcccaatg
attggcaaaaaaaaaaaaaaaaaaaaaaaaaaaaaaaaaaaaaaaaaaaaaaaaaaaaaaaaaaaaagaa
attaacacaaacaaaaaaatgaaaagacatctgGGCTTAGGGACtggaaaattaatattgttaaaatgtccata
ctacccaaagcaatctacagattcaatgtaattcttATACATATCCAAATGgattttttttacagaaatagaaaa
actaattccaaaattcatatgaaaacagTAGACCCCCAAATTGCCAAAGATTCTTAAGAAagcagaacaaagct
ggaggtatcatacTTTTTGCttgtaaaatatattacaaacctacagtaatcaaaacaatgtggtacTTGCATAA
AGACAGATGCATAAATCAATGGAAGAGAAGAGGGAGACCATAAATAAACCAATACATATACAGTCAACTGATCA
TCAACAAGggcatttaaaaaactgaatatcTACATGCAGAAGAGTAAAATGGAGCCTACactac

>SagImp_10289 Platy-1-8c_*Saguinus*, LS, inside L1MA2; Endo:ATTT/AA
acagtaagaatacatggacacagggaggggagcatcacacactgtggtctgttgaggggaataggggaggaac
agcaggtggtagggaggttggggagggataacatggggagaaatgccagatatccAGATATAGCTGATCGGGGA
TGGAGGCAtgaaaccacattgccatatatttacctatgcaacaatcttgcatgttcttcacatgtaccccagaa
cctaaagtgaaattatatatatatatatatatattttcttgtttttcataagagaacacttaagatctaccCTT
TTAGTGAGTTTCAAATATAAAACACTGTATTGTTGACtacagtcaccatgctgtacattagatctctagaAATT
ATTTGACTTGCATAACTTAAACTTAGTCCCGTTTGGCCAACATCATCTTATTACCCCATTTTctagcccttggc
aaccactgttctactctctgcttatgtgagtttgattattttaaatttcatgtaaggggcccaaagtggctccg
gccggatgtcatggcgcttgcgaaggcgaggccaagagttcaaggccaaccaacctcagcaaccttacaagctc
tcattgattggcaaaaaaaaaaaaaaaaaaaaaaaaaaaaaaaaaaaaaaaaaaaaaaaattcatg
taaaagtgagatcatgtagtatttgttttctgtgtttggCTTAATTTTCTCAGTATAATGTTCTCTAGGTTCA
TCGATGTCATCAGAGAAAaggcaggatttctttctttttttcaaatgaataatatttcattgtgtgtgtataca
cacaccacattttctttatccatgtaGATGTTGACAAACACTTAGATCATTTCCATATTTTGgctgttgtaaat
aatgctgcaatgaaggTGCAAATATTTCCTTGAAATAGtagttttttttgtatatatatgcagAACTGAGATTG
CTATATCACAaatggttctatttttaatttttgaaggaaTGTATTATACTGAAGAAATTAGATTCCTTTTACAT
TAGAGGAGCCTCCTAATTTTGCTGAGCCAACTTATGTTCTCACCAACAGtattttatcttttgtcttttt

>SagImp_12738 Platy-1-8c_*Saguinus*, LS, inside L1M1; Endo:TTTT/AA

```
AAGCAAGGACAAAACTATAAAgtattttgaactaaataaaataaaaatacatatcaaatttgtggaatTTCAC
TAAAGTAGTAGAAAGGAAAAacttacagcaataaatgcctcTATTAGGATATAAGAAAAGTTCCAAATAAAAAC
ACTCAGCTTTTACATTAAGAACCAAGAAacgaagagcaaattaaatccacaGAAGCAGAAGAAGCATAGTTGAA
AATTTTAGATTGAGTATAAATAGAGTAGAAAAtaggaaaacaatagagaaaaaaattaatgaaatgaaaagctG
GCTCTTTGGGAATATCAATAAAATTGAGAACCTGAAGGcacacagatgaagaaaatggaCATAAAACACTTACC
AATATCAGGAAGAAGACAGCTTGCATCACTACGGGCTATTCAAacgttaaaataaatgaatattttgaaCTACT
TTATGCCATTTCAACTTAGATAAAATGGCAAATTTGTTAAAAGACACAAATtattgggcccaaagtggctccgg
ccggatgtcgtggcgcttgcgaaggcgaggccaagagttcaaggccaaccaacctcagcaaccttacaagctct
cattgattggcaaaaaaaaaaaaaaaaaaaaaaaaaaaaaaaaaaaataaaaaaaaaaaaaaaaaaaaaaaaaa
aaaaaaaaaaaaaaaaaaaaaaaaagacacaaattattAAACTCATACAAGAAGAATTGATCATCTAAATAGCC
CTATACCTATTAAAGAAATCTAACTCATTGTTAAAAAATCTTCCCCTCCAGACCCAAATAGCTTTGCTAACAAG
TTATACCTAATatttaacaaagaaataatacaaattctaCACAAACTCTTTATAAAACTAAAGAGGAGAAAATA
TCTCTTAACTCATCCTATGAAGCCAGAAGTATCTTGACACTAACACCTGATAATGAcattaaaagaaaacatca
gataGTATTTTTCATAAACAAAGCTGCaaacattataaatataattttagaaaattgaatccaatcatattttt
aaaacatgacatGTCATGACCAAGTGGAGTGTATAAATTGGTTTGACATTTTACAACCAGTCAATGTCA
```

B. *S. imperator* intra-L1 Platy-1 elements from other *Saguinus* specific subfamilies

```
>SagImp_5561 Platy-1-8a2_Saguinus, LS, inside L1P1, -strand:rc;
Endo:TCTT/AT
agaaaagcattccatgctcatggtaaagaagaatcaatattgtgaaaatggccatactgccccaagtaatttat
agattcaatgctattcccatcaataTGGAACTGCAGAAAAGCCcggatagccaagacaatcctaagcaaaaaca
acaacaaaaaaacaacaacaacaaaaaaacaagctaCAGGCATCAaggtacctgacttcaaactatactacaag
gcttcagtaatcaaaacagccttgtactggtaccaaaacggatacatagaccaattgaacagcagagaggcctc
agaaacaatgcAACACATATACAatcatctgacctttgacaaacctgtcaaaaacaagcaacaaggaaaggatt
ccctatttaataaatggtgttgggaaaactggctagcaatatgcagaaagctgaaactagatctcttccttaca
ccttatataaaggtaactctagatggattaaagatttaaacataagacctaacactgaGGCTcacagtggctct
ggccagatgttgTGGTGCTTGTGGAGGTGAGGCCATgaattcaaggccaacctgagcaaccttacaagctctca
ttgattggaaaaaaagaaaagacctaacaccataaaaaccctagaagaaaatctaggcaataccattcaggaca
taggcatgggcaaggatttcatgactaaaccagaaaaagcaatggcaacaaaagccaaaattggcaaACAGGAT
CTacttaaacttaagagcttctgcacagcaaaagaaactaccatcagagtgaaccagcaaccaacagaatggga
aaaaattttttgcaatctacccatctgacaaagggctaatatccagaatatacaaggcacttaaagaaatttaca
agaaaaataaaaaacaaccacatcaaaaagtggatgaagactatcaacagacacttctcaaaagaagacattta
tgcagccaactaacataggaaaaaaagcccatcatcactggtcattagagaaatgcaaatcaaaattacattga
gataccatctcatgccagttagaatggtgatcattagaaaatctggagacaacagatgctggagag
```

```
>SagImp_1009 Platy-1-8a4_Saguinus; LS; inside L1PA10; -strand:rc;
Endo:TGTT/CT
Attgatcaagcagaagaaaggatatcagaggtggaagtccaacttaataaaataaaatgagaagacaagaatag
ggaaaaaaggataaaaaggaatgagtaaagtctccaagaaatatgggactatgtgaaaagacctaatctatgtt
tgataggtgtacctgaatgcgatggagagaatgaatccaagctggaaaatattgttcaggatattattcaggaa
aactttcccaacctagcaaagcaggacaatattcaaccccaggtaatacagagaacaccacaaagatattcctc
aagaagagcaaccccaaggcacataatcattagattcaccagggttgaaacaaaggagaaaattctaagggcag
ccagagagaaaggtcaggttacccaaaaagagaagtctatcagacttacagcagaatctctcagcagaaaccct
acaagccagaagagagtgggggctgatattcaacatccttaaagaacagatcttttgggcccacagtggctccg
gccagatgtcatggcacttgcgaaggcgaggccatgagttcaaggccaacctgagcaaccttacaagctctcat
tgattggcaaaacaaaaacaaaaacaaaaacaaaaacaaaaacaaaaaacaaaaaaacaaaaaacagatctttc
agtccagaatttcatatccagccaaactaagcttcacaactgaaggaaaaataaaatcttttatgaacaagcaa
gtactcagagattttattaccaccaggcctgttttacaagagcttctgagagaagcatagaaaggaacaaccag
tattagcctttctaaaaatataccaaaaagtaaagagcatcaacataaagaagaatttacatcaatgaatggat
aaaacacagtttacatcaaatggcagtaatcctaaatttaaatcgactaaatcccccaatcaaaagatacagcc
aaaacccaacggtatgctacatccagacccatttcacatgcaaggatacacaaagactcaaaacaaagggatgg
agaaagatttaccaaccaaatggagagcaaaaataaataaataaataaaaagcaggagttgcaat
```

>SagImp_12743 Platy–1-8a6_*Saguinus*, LS, inside L1P3; Endo: ACTT/AA
gatgtatagaaatgattgtgatttctgcacattgattttgtatcctgagactttgatgaaattgcttatcagtt
taaggagatttggggctgagatgataggatcttctaaatatacactcgtatcatctgcaaatggaggcaatttg
acttcttttcataattgaatgccctttatttttttttcttgcgtAATTGCTCttttagaacttccaatactata
ttgaataggagtggcaagagagggcatccttgtctagtgccagatttcaaagggaatgcttccagtttttgccc
atttagtataatattggctgtaggtctgtcgtaaatagcttttattattttgagatacgttccttcaatacctta
gtttattaagagtttttagcataaatgcggttgaattttgtctaacgccttctctgcatctattgagataatca
agtggttttttgtctttggttttgtttatgtgatggattaagtttatagacttgccgggcccaaagtggctctgg
ccggatgtcgtggcacttgcgaaggcgaggccatgagttcaaggccaacctgagcagtcttacaagctcttatt
gatagggaaaaaaaaaaaagtttatagacttgcctatgttgaaccagcctggaatgaagcctatttgatcataa
tggataagctatttgatgtgctgttgcaatcagtttagcagtattttagtgaagatttttttgcatgtatgttt
atcagggatattggcctgaagttttcttttcttgttgagtctctgctgggttttggtatcaggatgatgttggt
ctcataaaatgatttggggaggattccctcttcttggattgtttggaatagtttcagaaggaatagtacaagct
tctctttgtatgtctggtagaattcggttgtcaacccatctgaacctgggcttttattggttggtaggcttta
atCACCACCTCAAAtttagcccttgttattgatctattcagggcttcaacttcttcctggtttaggcttgaaag
ggTGCAAGTGatcaggaatttatacatttcttccaggtttagtaTTCtatgtgcagagttgtttg

>SagImp_8039 Platy-1-8a5b2_*Saguinus*, LS, inside L1PA17; Endo:TATT/CT
actcccaacctcaagcaatctgcccacctcagtttcccaaagtgctgggattacaggcatgagccatcacctgG
CTGAGATTTTTCTTTCAAGATGTATGTGTTCACTACTATAAACTtcctctgtttttttattttagattcagaggg
tatatgtacacatttgtcaaataaatatattgcatACTGGTGAGGTTTGGCTTCTAGTGAACCTGTCTCTGAAA
TAGTAAACATTGTGCtcaacagatttcttttttttttgaggcatgttcttgctctgtcaccctagctgttgtg
cagtggcatgatcacagctcactatcaATAGGTAATTTTGTGACCATCAACCTCCTTCCAATCACTCCTTTTGG
AGTCCCAATatctattgtttctatttttatattctgttgtgctcattgtttagctctcgcttgtaagtaagaac
atgtggtatttgatttctgtttctgagttatttcacttagaataatggcccccagggggcccaaagtggctcc
agccggatgtcgtggcacttgcaaaggtgaggccaagagttcaaggccaacctcagcaaccttaaaagctctca
ttgattggcaataataataataataatggcccccagatccatccatgttactgcatagagcatggttttatttt
ttatggcttcatagtattccatgctgtacatAAACCACATGTAAAAATCCAGTCcactgttaatggacatttag
gttaattccacgACTTTACTATTATGAGTAGGAATGTAAGACACATATGAGTGAAAGtcacttttttaatataat
tatttatttttttctttcaggtaGATGCCCAGTAGTTTTATAATTGGGTTAAATAGTGGCtctatttttaatgct
ttgagaaatctctatattGTTTTTTTATAACAGTTGTGCTCACTCACATTTCCACCACCAGTGAATAGGCATTC
ATTATACTCTGCATCCTCAAAAACATTTGTTGTATTTGTAGTACTAGCCATTCTGtgtaagatgatatcttatt
gtggtttgtttgtttttgagatggagtctcattctgtccctcaggctggagtgcagtagtataatct

>SagImp_2651 Platy-1-8b_*Saguinus*, LS, inside L1M1, -strand:rc;
Endo:TTTT/AA
gggcaacacagggagaccccatctctacaagataaatattttttaattagctggatatggtggcacaagcttgt
ggtcccagcgactcaggaagctgagctgggaggatcacttgaacccaagaggtcaaggctgcagtgagccttga
ttgtgaAACTGAatccaccctggatgacagatCAAGATTCCATcttacagagctacagtaaccaaaacagcatg
gtactgacataaaaacagaggAATGAAACAGAATGGAGGACACAGAAATGAATCCATACATCTACAAAAATGAA
TCCACTCATTTTGACAAAGGTACTAAGAACACatactggggaaaagacagtttcttcaataaatggtgcttgga
aaactgaatatccatacacagaagggagaaactagaccctatctctcaccatatataaaaatcaaattaaaatg
gattaaagatctaaatctAGGACCTGAAACTATCaaactattaaaagaaaacactgggggcccaaagtggctcc
ggtcgGATgccgtggcacttgcgaaggcgaggccaagagttcaaggccaaccaatcttacaatctcccaatgat
tggcaaaaaaaaaaaaaaaaaaaaaaaagaaaacactgaagaTCTCTCTAGGACATTAGTCtgggcaaatattt
cttgagtagtACCCCAAAAGCACTggaaatcaaagcaaaaatgcaCAActaggatcacatcaagttaaaaagct
tctgcacagtgaaggaaacaatcagcaaagtgaagaagcaactcacagaatgggagaaaatatttacaaaattt
gCAAACTGtctatctgacaagggattagtAAGAATATGTAAGGAGTTCTAATAATAAGataaaaatctgatttt
aaaatgggcaaaagatgggaatagacatttctcaaaagctATACAGAAGGCAAACAGGTATAAGAAAAGGTGCT
CcatatcactgatcatcagacaaatgcaaatcaaaactacagtaagATATTATGTTAGCACACgtaaaatgact
tttatccaaaagacagggaacaacaaatgctggcaaggatgtggagaaaagggaacccttg
